# A Functional Binding Domain in the Rbpr2 Receptor Is Required for Vitamin A Transport, Ocular Retinoid Homeostasis, and Photoreceptor Cell Survival in Zebrafish

**DOI:** 10.3390/cells9051099

**Published:** 2020-04-29

**Authors:** Ashish K. Solanki, Altaf A. Kondkar, Joseph Fogerty, Yanhui Su, Seok-Hyung Kim, Joshua H. Lipschutz, Deepak Nihalani, Brian D. Perkins, Glenn P. Lobo

**Affiliations:** 1 Department of Medicine, Medical University of South Carolina, Charleston, SC 29425, USA; solankia@musc.edu (A.K.S.); su@musc.edu (Y.S.); kims@musc.edu (S.-H.K.); lipschut@musc.edu (J.H.L.); nihalani@musc.edu (D.N.); 2 Glaucoma Research Chair, Department of Ophthalmology, College of Medicine, King Saud University, Riyadh 11411, Saudi Arabia; akondkar@ksu.edu.sa; 3 Department of Ophthalmic Research, Cole Eye Institute, Cleveland Clinic, Cleveland, OH 44195, USA; fogertj@ccf.org (J.F.); perkinB2@ccf.org (B.D.P.); 4 Ralph H. Johnson VA Medical Center, Division of Research, Charleston, SC 29420, USA; 5 Department of Ophthalmology, Medical University of South Carolina, Charleston, SC 29425, USA

**Keywords:** retinol binding protein 4 receptor 2, RBP4, Rbpr2, STRA6, all-*trans* retinol transport, photoreceptor cell, vision, retinoids, zebrafish

## Abstract

Dietary vitamin A/all-*trans* retinol/ROL plays a critical role in human vision. ROL circulates bound to the plasma retinol-binding protein (RBP4) as RBP4-ROL. In the eye, the STRA6 membrane receptor binds to circulatory RBP4 and internalizes ROL. STRA6 is, however, not expressed in systemic tissues, where there is high affinity RBP4 binding and ROL uptake. We tested the hypothesis that the second retinol binding protein 4 receptor 2 (Rbpr2), which is highly expressed in systemic tissues of zebrafish and mouse, contains a functional RBP4 binding domain, critical for ROL transport. As for STRA6, modeling and docking studies confirmed three conserved RBP4 binding residues in zebrafish Rbpr2. In cell culture studies, disruption of the RBP4 binding residues on Rbpr2 almost completely abolished uptake of exogenous vitamin A. CRISPR-generated *rbpr2-*RBP4 domain zebrafish mutants showed microphthalmia, shorter photoreceptor outer segments, and decreased opsins, which were attributed to impaired ocular retinoid content. Injection of WT-Rbpr2 mRNA into *rbpr2* mutant or all-*trans* retinoic acid treatment rescued the mutant eye phenotypes. In conclusion, zebrafish Rbpr2 contains a putative extracellular RBP4-ROL ligand-binding domain, critical for yolk vitamin A transport to the eye for ocular retinoid production and homeostasis, for photoreceptor cell survival.

## 1. Introduction

Dietarily or maternally derived vitamin A (all-*trans* retinol) and its metabolites (retinoids) regulate many biological and cellular processes, including metabolism, differentiation and proliferation, and is essential for embryonic development, immune function, reproduction, and vision in humans [1,2,3,4,5,6,7,8,9,10,11,12,13,14]. Vitamin A deficiency or excess during development affects many vertebrate organs, including the eye [4,5,15,16,17,18,19,20,21]. The most well-known early effects of vitamin A deficiency in humans is night blindness [22,23], while prolonged vitamin A deficiency during pregnancy has been linked to increased microphthalmia, childhood mortality and morbidity, and photoreceptor cell death and progressive vision loss in adulthood [24,25,26,27]. Given the many biological functions of retinoids, the embryo’s dependence on vitamin A for survival and development, and cellular toxicity associated with vitamin A overload demand a specific and stable mechanism of vitamin A transport into cells [28,29].

Dietary vitamin A is the precursor for at least two critical metabolites, all-*trans* retinoic acid (a*t*RA) and 11-*cis* retinaldehyde (11-*cis* RAL) [16]. Evolution’s choice of dietary vitamin A as a precursor for the vital signaling molecule (a*t*-RA) for retinal cell development and maintenance, and the essential visual chromophore (11-*cis* RAL) in photoreceptors, triggered selective pressure to advance an efficient system of transporters for dietary vitamin A uptake and storage [5,10,11,28,29]. All-*trans* retinol (ROL) is the main transport form of dietary vitamin A in the blood. During transport virtually all ROL is bound to plasma retinol-binding protein (RBP4). RBP4 delivers ROL from the liver, the main organ of storage, to distant organs that need vitamin A such as the eyes, brain, lungs, kidneys, placenta, and other peripheral organs [2,5,11,28,29]. Here, although RBP4 solubilizes ROL in the circulation the complex of RBP4-ROL (holo-RBP4) cannot diffuse through the cell membrane and therefore requires a membrane receptor to facilitate the transport of ROL into cells.

The cell membrane surface receptor for RBP4 in the eye has previously been identified as the multi-transmembrane domain protein receptor STRA6 (Stimulated by retinoic acid 6) [28,29,30,31]. STRA6 binds to RBP4 with high affinity and specificity, and this facilitates cellular uptake and intracellular transport of ROL from holo-RBP4 into the retinal pigmented epithelium (RPE). However, STRA6 is not expressed in the liver, intestine, lungs, and other peripheral tissues proposed to express a membrane receptor that is mediates systemic uptake and peripheral tissue storage of food-derived ROL [2,5,32,33]. Likewise, while we and others have shown that the scavenger receptor class B type 1 (SR-B1) protein is involved in cellular uptake of dietary pro-vitamin A carotenoids for ROL production, SR-B1 is not involved in RBP4-ROL transport [34,35,36,37]. Therefore, since there is high-affinity RBP4-ROL transport in the liver, intestine, and other peripheral tissues known to acquire and store vitamin A from its plasma bound form, this implies the existence of a second RBP4-ROL transporter [2,5].

Previously, the existence of a second RBP4 receptor expressed in tissues lacking STRA6 was postulated [2,5], and in 2013, the retinol binding protein 4 receptor 2 or stimulated by retinoic acid 6 like protein (*RBPR2/STRA6L*) was molecularly identified [2]. Hence, the RBPR2 gene product that is highly expressed in mouse liver, lung, and intestine could function as the second RBP4-ROL transporter and could therefore contribute to whole-body retinoid homeostasis and for ocular retinoid production in the support of vision [2]. Rbpr2 shares structural homology with STRA6 and is predicted to have 9–11 transmembrane domains like STRA6. Both the zebrafish *rbpr2* and mouse *Rbpr2* genes contain several short amino acid segments with >50% amino acid identity to *STRA6*, but with <22% overall amino acid identity [2,33].

Based on our recent observations in zebrafish and results reported in the literature, we hypothesize that Rbpr2 facilitates systemic and peripheral tissue uptake of dietary vitamin A for whole-body retinoid homeostasis in support of organ development. Our hypothesis is supported by recent studies in cell culture where zebrafish Rbpr2 localized to cell surface membranes and promoted RBP4-ROL uptake [32,33]. Two *rbpr2* mutant zebrafish lines, each resulting in Rbpr2 deficiency, exhibited smaller eyes/microphthalmia visible from early embryonic developmental stages, and multi-organ malformations at late larval stages, consistent with phenotypes previously associated with vitamin A deficiency [33]. These observations led us to hypothesize that the Rbpr2 transporter likely contains conserved RBP4 binding domains for ROL uptake and this physical protein-protein interaction plays a critical in vivo role in systemic ROL transport.

In this study, we used two complementary approaches to further study the structure and function of Rbpr2 for systemic retinol uptake from RBP4. The first approach was to mutate the amino acid residues in the proposed RBP4 binding domain in zebrafish Rbpr2 and systematically analyze their membrane trafficking patterns and extracellular ROL uptake properties in vitro. The second approach took advantage of the CRISPR/Cas9 genome editing technology to generate zebrafish mutants targeting the proposed RBP4-ROL binding residues in Rbpr2. Since the eye is the human organ most sensitive to vitamin A deficiency during development and in adulthood, we used retinal phenotypes and quantified ocular retinoid content, as functional readouts to study the effects of loss of the proposed RBP4-ROL binding domain in Rbpr2, for vision. The studies reported here demonstrate that the second vitamin A transporter Rbpr2 in zebrafish, like STRA6, contains a functional RBP4 binding domain that is critical for yolk vitamin A delivery to the vertebrate eye during development.

## 2. Materials and Methods

### 2.1. Materials

All chemicals, unless stated otherwise, were purchased from Sigma-Aldrich (St. Louis, MO, USA) and were of molecular or cell culture grade quality.

### 2.2. Homology Modeling and Molecular Docking

The online server SWISS-MODEL (http://swissmodel.expasy.org/) was used to generate homology-based models of zebrafish Rbpr2 and human STRA6 [38,39]. The model with maximum coverage and lowest Z score for each protein was selected for further studies. The SWISS-MODEL server was used for 3D structure prediction and template selection, template was selected based on maximum similarity or identity with sequence. The template selected (by the online server SWISS-MODEL) was the crystal structure of zebrafish Stra6 receptor for retinol (PDB ID 5sy1, CHAIN B), which showed nearly 44% sequence identity for human STRA6; and nearly 22% sequence identity for zebrafish Rbpr2. The structure for RBP4 was obtained from the PDB database (RSCB PDB ID: 2wqa, Chain E). Accuracy and quality of the predicted models were analyzed by performing RAMPAGE for Ramachandran plot analysis. The best selected models were based on the total number of residues of over 90% in the most favored regions. The models generated were used for docking studies to analyze the protein-protein interactions employing the online data-driven docking program HADDOCK [38]. HADDOCK requires a set of ambiguous interaction restraints (AIRs) at the binding interface that are divided into “active” and “passive” categories, where active residues are those directly implicated in binding from experimental data, and passive residues are their near neighbors. The docking process included a rigid body energy minimization step. Residues S325, Y326 and L327 in human STRA6 and residues S268, Y272 and L273 in Rbpr2 were assigned as active residues for interaction as they have previously been identified to be part of an essential binding domain for RBP4-ROL [2,9,28,29]. The residues between 8 and 12 Å from these three residues were defined as passive. HADDOCK clustered 187 structures in 11 cluster(s), which represents 93.5% of the water-refined models HADDOCK generated for RBP4-STRA6. HADDOCK clustered 138 structures in 12 cluster(s), which represents 69.0% of the water-refined models HADDOCK generated for RBP4-Rbpr2. The top cluster, with the minimal haddock scores of −85.6 +/− 2.0 and −81.4 +/− 3.1 and the lowest Z-scores of −1.6 and −1.4 for RBP4-Rbpr2 and RBP4-STRA6, respectively, was selected for analysis (Table 1).

### 2.3. Cloning of Zebrafish Rbpr2 cDNA

The full-length zebrafish *Rbpr2* cDNA product generated by PCR was cloned in frame into the pCDNA3.1 V5/His TOPO vector (Invitrogen, Carlsbad, CA, USA) as previously described [32,33].

### 2.4. Site-Directed Mutagenesis and Generation of Stable Cell Lines Expressing Mutant Rbpr2 binding Residue Recombinant Proteins

Mouse NIH3T3 cells obtained from American Type Tissue Culture (ATCC-1658) were maintained in high-glucose DMEM supplemented with 10% FBS and 1% penicillin-streptomycin sulfate, and cultured at 37 °C with 5% CO_2_. To generate NIH3T3 cells constitutively expressing zebrafish Rbpr2, parental NIH3T3 or NIH3T3/LRAT expressing cells were transiently transfected with the V5-tagged pRbpr2 plasmid, as previously described [32,33]. To confirm stable integration of the *rbpr2* gene and expression in these cells, we isolated total protein from each clone and subjected them to Western blot analysis. A V5-primary antibody (Sigma/Millipore, Cambridge, MA, USA) was used to detect the V5-tagged Rbpr2 protein. The WT-Rbpr2 plasmid was used as a template and mutagenic Rbpr2 primer pairs were used to engineer each of the RBP4 binding residue mutants by in vitro site-directed mutagenesis (Quick Change II XL: Stratagene/Agilent, Santa Clara, CA, USA), as previously achieved [32,33,40]. Appropriate construction of the WT-Rbpr2 and mutant-Rbpr2 plasmids were verified by DNA sequence analysis of both strands using pCDNA3.1 vector primers (Genewiz, Research Triangle Park, NC, USA).

### 2.5. Indirect Immunofluorescence and Confocal Microscopy

Stable cell lines were grown on coverslips and fixed in a freshly prepared mixture of 4% paraformaldehyde in 1X PBS (137 mM NaCl, 2.7 mM KCl, 10 mM sodium phosphate dibasic, and 2 mM potassium phosphate monobasic, pH 7.4) for 30 min at room temperature and processed as previously described [32,33,40]. Subcellular localization of the recombinant V5-tagged zebrafish Rbpr2 in NIH3T3 cells was achieved by exposure to the anti-V5 primary antibody followed by anti-rabbit conjugated Alexa 488 secondary antibody staining (Invitrogen, Carlsbad, CA, USA). Cells were examined under a Zeiss LSM 510 UV Meta confocal microscope with an HCX Plan ×40 numerical aperture 1.4 oil immersion objective lens (Zeiss, Jena, Germany). Images were acquired with the Zeiss confocal software, version 2.0. All experiments were carried out in triplicate. Approximately 50–70 cells from 4–5 fields were imaged and counted per experiment.

### 2.6. RBP4 binding and Retinol Uptake Studies

Human RBP4 cDNA cloned into the pET3a bacterial expression vector was used to express RBP4 in *E.Coli* as previously described [31,33]. Stable NIH3T3 cells expressing either WT-Rbpr2 or WT-Rbpr2/Lrat or individual Rbpr2 mutants/Lrat were plated in 10 cm dishes. Cells were grown to 70% confluency, washed thrice with 1x PBS and incubated for 1 h in serum-free medium, at which point [^3^H]retinol-RBP4 was added for 60 min. Cells were washed thrice with 1x PBS and lysed in PBS containing 1% Nonidet P-40. Lysates were homogenized and transferred to scintillation tubes for scintillation counting. Parental NIH3T3 and NIH3T3/LRAT cells incubated with [^3^H]retinol-RBP4 served as controls.

### 2.7. RBP4 binding to Rbpr2 in Co-immunoprecipitation Assays and UV Crosslinking

Co-immunoprecipitation experiments were performed with exogenously applied holo-RBP4 protein in stable NIH3T3/LRAT cells expressing V5-tagged WT-Rbpr2 or individual Rbpr2 mutants. The amine-reactive group on the SANPAH crosslinker (ThermoFisher, Waltham, MA, USA) was covalently linked to the purified His-RBP4. The His-RBP4 and crosslinker conjugate was then added to stable cells and UV crosslinked as described previously [9].

### 2.8. Animal Study Approval

All experiments on zebrafish were approved by the Institutional Animal Care and Use Committee (IACUC: Zebrafish Protocol #3489 and #00774) of the Medical University of South Carolina and the Cleveland Clinic, and were performed in compliance with the ARVO Statement for the Use of Animals in Ophthalmic and Vision Research.

### 2.9. Zebrafish Strains and Maintenance

Commercial *rbpr2* mutant zebrafish lines (*rbpr2*^sa107076^ and *rbpr2*^sa32616^) were obtained from the Zebrafish International Resource Center (ZIRC). The transgenic *Tg(XlRho:EGFP)^fl1^*, *Tg(-3.2gnat2:EGFP)^ucd1^/Tg(3.2TalphaCP:EGFP)*, WT (strain AB/TU) zebrafish lines, and others used in this manuscript were bred and maintained under standard conditions at 28.5 °C [41,42]. Collected embryos were maintained in embryo medium (15 mM NaCl, 0.5 mM KCl, 1 mM CaCl_2_, 1 mM MgSO_4_, 0.15 mM KH_2_PO_4_, 0.05 mM NH_2_PO_4_, 0.7 mM NaHCO_3_) at 28.5 °C. Morphological features were used to determine the stage of the embryos in hours (hpf) or days (dpf) post fertilization [41].

### 2.10. Zebrafish Immunohistochemistry and Fluorescence Imaging

Using established protocols, 2–6 days post fertilization (dpf) zebrafish larvae were fixed in 4% paraformaldehyde buffered with 1X PBS for 2 h at 4 °C [42]. After fixation, samples were washed in 1X PBS and cryoprotected with 30% sucrose/1X PBS for 48 h at 4 °C. Samples were oriented in freezing molds in 100% Tissue-Tek OCT (ThermoFisher) and stored at −20 °C until sectioning. Cryosections (10 µm) were cut and dried onto frost-free slides at RT overnight. Slide edges were lined with a hydrophobic marker (PAP pen) and washed with PBS before blocking for 1–2 h at RT. Blocking solution (1% BSA, 5% normal goat serum, 0.2% Triton-X-100, 0.1% Tween-20 in 1X PBS) was applied for 2 h in a humidified chamber. Primary antibodies were diluted in blocking solution as follows: anti-1D4/Long-cones (1:250, Abcam, Cambridge, MA, USA), PNA-488 (1:2000, Molecular Probes, Eugene, OR, USA), anti-Red/Green cone opsin (1:500; Sigma/Millipore, St. Louis, MO, USA), and 4′,6-diamidino-2-phenylendole (DAPI; 1:5000) was used to label nuclei. All secondary antibodies (Alexa 488 or Alexa 594) were used at 1:5000 concentrations (Molecular Probes, Eugene, OR, USA). Optical sections were obtained with a Leica SP8 confocal microscope (Leica, Germany) and processed with the Leica Viewer software.

### 2.11. Generation of Zebrafish rbpr2 mutants using CRISPR/Cas9 

The program Chopchop (https://chopchop.rc.fas.harvard.edu/) was used to design guide RNAs oligonucleotides (gRNA) targeting the zebrafish Rbpr2 exon 8, to genetically disrupt the proposed “SYL” RBP4 binding domains [2,43,44,45]. CRISPR-gRNA contained a 20-base pair (bp) target sequence with a protospacer adjacent motif (PAM) site. Guide RNAs (100 pg) were co-injected with zebrafish codon optimized Cas9 mRNA (150 pg) into 60–70 wild-type (WT) one-cell stage embryos. At 6 months of age, five adult F0 *rbpr2*-mosaic mutant fish were outcrossed with a wild-type (WT) partner. The resulting F1 embryos were genotyped at 3 months of age via fin clipping and by *Hyp166II* restriction enzyme digestion of PCR products encompassing the SYL-RBP4 target sequence in Rbpr2. Disruption of the SYL-RBP4 binding domain in F1 heterozygote *rbpr2* mutants was confirmed by partial *Hyp166II* digestion and by sequencing. To negate any off-target effects caused by the CRISPR targeting, the confirmed F1 generation heterozygous *rbp2*-RBP4 mutant carriers were outcrossed for 2 generations with either the *Tg(-3.2gnat2:EGFP)^ucd1^/Tg(3.2TalphaCP:EGFP)* annotated as Tg:TαC-GFP or the *Tg(XlRho:EGFP)^fl1^* annotated as Tg:XOPS-GFP zebrafish lines. The Tg:TαC-GFP zebrafish line expresses soluble GFP driven by the cone specific transducin α-GFP in cone photoreceptor cells only [46], while the Tg:XOPS-GFP zebrafish line expresses soluble GFP driven by Rhodopsin in rod photoreceptor cells only [46,47]. F3 generation heterozygous *rbpr2* mutant carriers on the Tg:GFP backgrounds (annotated as *rbpr2^fs-^*^muz99^;Tg:TαC-GFP or *rbpr2^fs-^*^muz99^;Tg:XOPS-GFP) were re-confirmed by sequencing of genomic DNA from fin-clips.

### 2.12. rbpr2^fs-muz99^ Mutant Rescue Experiments Using WT or Mutant-rbpr2 mRNA

For rescue experiments of *rbpr2^fs-muz99^* zebrafish mutants, capped and polyadenylated mRNA of wild-type *rbpr2* (WT) or sequence-mutated zebrafish *rbpr2* (S268 and Y272P) was synthesized in vitro using the mMESSAGE mMACHINE kit (Ambion, Austin, TX) and Poly(A) Tailing Kit (ThermoFisher). Two doses of *rbpr2*-WT or mutant *rbpr2*-mRNA (low: 150 pg) or (high: 250 pg) were injected using a Sutter Instruments microinjector into ~100 embryos at the one or two-cell development stage. At the 5.5 dpf stage, twenty randomly selected injected larvae were imaged and then individually genotyped by direct sequencing as outlined above. Rescue experiments were repeated twice with a fresh preparation of capped and polyadenylated WT or mutant-*rbpr2* mRNA.

### 2.13. All-*Trans* Retinoic Acid Rescue Experiments in rbpr2^fs-muz99^ Mutant Zebrafish

Exogenous applied all-*trans* RA was dissolved in DMSO and applied at two different does (0.2 μM or 0.5 μM) to the fish water containing embryos (from heterozygous *rbpr2^fs-muz99^* parents) at the 40% epiboly stage, just before gastrulation begins. Control embryos were incubated with the vehicle only (0.1% DMSO) and showed no rescue of phenotype. Twenty-four larvae were imaged between 5–5.5 dpf and genotyped. Experiments were repeated twice. 

### 2.14. Apoptosis/TUNEL Assay

Frozen sections from retinas of wild-type and *rbpr2^fs-muz99^* mutants were stained using an in situ Cell Death Detection Kit, Fluorescin (Sigma/Millipore, Cambridge, MA, USA) according to the manufacturer’s instructions. The In situ Cell Death Detection protocol is based on the detection of single- and double-stranded DNA breaks (TUNEL technology: TdT-mediated dUTP-X nick end labeling) that occur at the early stages of apoptosis. Retinal sections were fixed and permeabilized. Subsequently, the retinal sections were incubated with the TUNEL reaction mixture that contains TdT and fluorescin dUTP. The apoptotic cells in retinal sections were photographed using confocal microscopy (Leica SP8, Germany).

### 2.15. Transmission Electron Microscopy (TEM)

Control and mutant zebrafish larvae at the indicated development time-points were fixed in a solution containing 2% Paraformaldehyde/2.5% Glutaraldehyde (buffered in 0.1M Cacodylate buffer) overnight at 4 °C. Samples were then rinsed in buffer (0.1M Cacodylate buffer). Post-fixative 2% OsO4/0.2M Cacodylate buffer 1 h at 4 °C, followed by 0.1M Cacodylate buffer wash. Samples were dehydrated through a graded ethanol series and then embedded in EmBed 812 (EM Sciences). The cured blocks were cut at 0.5 microns and stained with 1% toluidine blue to orient the blocks to the specific cell types wanted. The blocks were then trimmed to the specific size needed for ultrathin sectioning. The blocks were cut at 70 nm and gathered on 1 micron grids. The grids were air dried and then stained with uranyl acetate (15 min) and lead citrate (5 min) each and rinsed in-between each stain. They were then allowed to dry and imaged with a JEOL 1010, and images taken with a Hamamatsu camera and software. All samples were processed by the Electron Microscopy Resource Laboratory at the Medical University of South Carolina as previously achieved [33,48].

### 2.16. Western Blot Analysis and Densitometry

Total protein from cells or zebrafish larvae (*n* = 15 per genotype), was extracted using the M-PER protein lysis buffer (ThermoScientific, Beverly, MA) containing protease inhibitors (Roche, Indianapolis, IN, USA). Approximately 25 μg of total protein was then electrophoresed on 4–12% SDS-PAGE gels and transferred to PVDF membranes. Membranes were probed with primary antibodies against β-Actin or Gapdh (1:10,000, Sigma/Millipore, Cambridge, MA, USA), anti-V5 (1:1000, Sigma/Millipore, Cambridge, MA, USA), anti-Lrat (1:1000; Abcam, Cambridge, MA, USA), anti-Rhodopsin (1:1000, Sigma/Millipore, Cambridge, MA, USA), anti-PARP1 (1:2000; Cell Signaling, Danvers, MA) and anti-Red/Green cone opsin (1:1000, Sigma/Millipore, Cambridge, MA, USA) in antibody buffer (0.2% Triton X-100, 2% BSA, 1X PBS) [33,40,48,49]. HRP conjugated secondary antibodies (BioRad, Hercules, CA, USA) were used at 1:10,000 dilution. Protein expression was detected using a LI-COR Odyessy system and relative intensities of each band were quantified (densitometry) using Image J Software version 1.49, and normalized to the loading control.

### 2.17. Quantitative Real-Time PCR

RNA was obtained from heads of 5.5 dpf larvae, and isolated using Trizol reagent, and processed as described previously [33,35,36]. One microgram of total RNA was reverse transcribed using the SuperScript II cDNA Synthesis Kit (Invitrogen, Eugene, OR). Quantitative Real-Time PCR (qRT-PCR) was carried out using SYBR green 1 chemistry (BioRad, Hercules, CA). Zebrafish gene specific primers pairs for *rbpr2* (forward 5′-TCAGACTGAGAGTGTGTTTAC-3′ and reverse 5′-TACTGGCGGTGGTTTCATGACCT-3′), *aldh1a1* (forward 5′-TTCAACGTAGACTATGTAGAAAA-3′ and reverse 5′-AGCGACTGCTTTTTCCACA-3′), *aldh1a2* (forward 5′-CATTTTTGCAGATGCTGA TTT TG-3′ and reverse 5′-CAAAGATACGGGAACCAGCAGT-3′), *cyp26a1* (forward 5′-ATAAAGACGGACGAGCAAGA-3′ and reverse 5′-TCGTCATCTTGAATTTTCTT-3′), *lrat* (forward 5′-CGCGTACGGAGCTCCGATTC-3′ and reverse 5′-AACTCACCTTGTCGGTCTGC-3′), *dhrs3a* (forward 5′-GTCGGGGATGTCACCATTCTT-3′ and reverse 5′-ATTTGTCTCTTACCCAGAACT-3′), *rpe65* (forward 5′-GTTTTTCTCATATTTTAAGGG-3′ and reverse 5′-CTTTTTTAGCGTTTCCAGAGTG-3′). Retina gene expression was normalized to 18S ribosomal RNA expression (forward 5′-TCGCTAGTTGGCATCGTTTATG-3′ and reverse 5′-CGGAGGTTCGAAGACGATCA-3′). Samples for qRT-PCR experiments were assayed in triplicate using the BioRad CFX96 Q-PCR machine. Each experiment was repeated twice (*n* = 6 reactions for each gene) using newly synthesized cDNA. The ΔΔCt method was employed to calculate fold changes.

### 2.18. HPLC Analysis for Retinoids

Extraction of retinoids and HPLC analysis were carried out as previously described [35,36,49,50]. For quantification of the molar amounts, peak integrals were scaled with defined amounts of reference substances [31,49,50]. To determine the retinoid content of larval eyes, the eyes of dark-adapted larvae were removed by hand dissection with a scalpel under red safety light.

### 2.19. Statistical Analysis

Data are expressed as means ± standard deviation by ANOVA in the Statistica 12 software (StatSoft Inc., Tulsa, OK, USA). Differences between means were assessed by Tukey’s honestly significant difference (HSD) test. *p*-values below 0.05 (*p* < 0.05) were considered statistically significant. For Western blot analysis, relative intensities of each band were quantified (densitometry) using the Image J Software version 1.49 and normalized to the loading control β-Actin. QRT-PCR analysis was normalized to 18S RNA, and the ΔΔCt method was employed to calculate fold changes. Q-RTPCR data are expressed as mean ± standard error of mean (SEM). Statistical analysis was carried out using PRISM 8 software-GraphPad. For Figures 4A, 8B–D, 9B, and 10C, additional statistical analysis of data using the Mann–Whitney *U* test are presented in the corresponding figure legends. The data between groups were considered significant when *p* < 0.05 (medians are provided in Box-Whisker Plots in Supplementary Figures S13–S16, respectively).

## 3. Results

### 3.1. Zebrafish Rbpr2 Contains Consensus RBP4 Binding Residues

**Figure 1:**
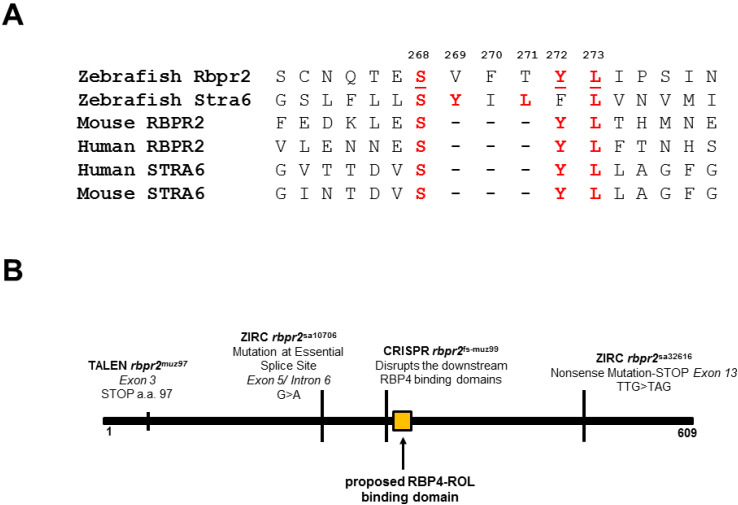
Proposed RBP4 binding residues on zebrafish Rbpr2 for retinol transport. (**A**) A proposed RBP4 binding domain (SYL) in Rbpr2 is conserved among species. This RBP4 binding domain occurs in exon 8 of Rbpr2. Overall amino acid identity between STRA6 and Rbpr2 among species is <22% based on this alignment. (**B**) Schematic representation of zebrafish Rbpr2 cDNA. Location of mutations in *rbpr2* mutant zebrafish lines generated or acquired previously in relation to the proposed RBP4 binding domain are shown.

Our recent data, and the results in the literature, suggest that Rbpr2 has high binding affinity for RBP4, and this facilitates the systemic uptake of protein bound vitamin A/all-*trans* retinol/ROL (RBP4-ROL) into tissues not expressing STRA6 [2,32,33,51]. This implies that conserved RBP4 binding residues/domain must exist on Rbpr2 to facilitate this process [2,33,43]. Therefore, it is of importance to identify and functionally test the proposed RBP4 binding residues on Rbpr2 for vitamin A transport. Alignment and comparison of zebrafish, mouse and human RBPR2 protein sequences with human, zebrafish and mouse STRA6 protein sequences revealed <22% overall amino acid identity but several short amino acid segments with >50% amino acid identity [2,43] (Figure 1A). The conserved amino acid sequences between Rbpr2 and STRA6 suggests analogous roles for these residues in the function or structural integrity of these two proteins [2,9,10,28]. Interestingly, a consensus three amino acid proposed RBP4 binding domain (Serine-Tyrosine-Lysine) in human and mouse STRA6 is also partially conserved in the zebrafish Rbpr2 sequence (Figure 1A). The proposed RBP4 binding site corresponds to amino acids Serine268, Tryosine272 and Lysine273 in exon 8 of the zebrafish *rbpr2* gene and lies downstream of the two mutations (*rbpr2*^muz97^ and *rbpr2*^sa10706^; *rbpr2* mutant zebrafish lines) previously characterized by us (Figure 1B) [33]. 

### 3.2. Homology Modeling and Molecular Docking Analysis Confirm Importance of a Proposed RBP4 Binding Domain in Rbpr2 for Vitamin A Transport

**Figure 2:**
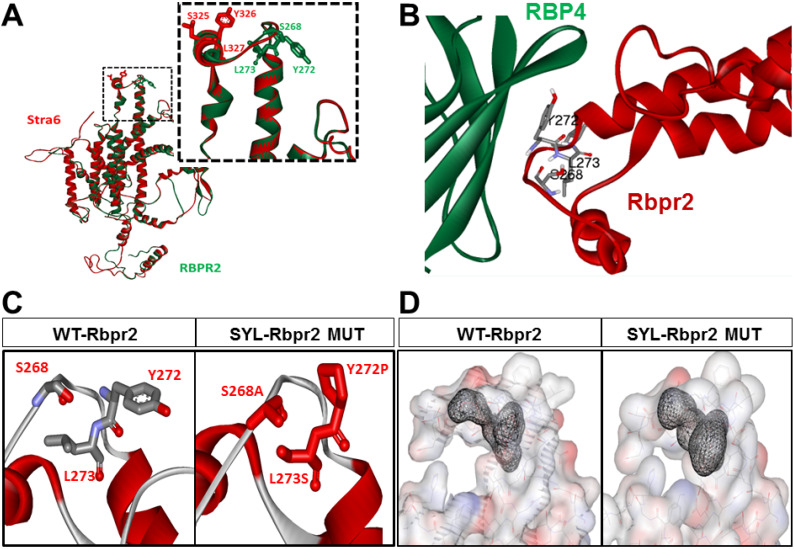
Homology modeling and molecular docking analysis of Rbpr2-RBP4 protein interaction. (**A**) Structural overlay of the homology models generated for human STRA6 (red) and zebrafish Rbpr2 (green) using SWISS-MODEL server. As shown, Rbpr2 and STRA6 share significant structural homology with a conserved three amino acid “SYL”-RBP4 binding domain (large box). (**B**) Rbpr2-RBP4 protein-protein interaction model is shown for zebrafish Rbpr2 and human RBP4 in ribbon view. (**C**,**D**) Disruption of any of the individual SYL-RBP4 binding residues in the extracellular loop of Rbpr2 is predicted to reduce the free energy gap between native and mutated protein states and affect proper extracellular RBP4 binding and intracellular retinol transport.

As previously used by us, the online server SWISS-MODEL (http://swissmodel.expasy.org/) was used to generate homology-based models of zebrafish Rbpr2 and human STRA6 [9,10,39,40,43] and the structural overlay of the models generated for human STRA6 (red) and zebrafish Rbpr2 (green) are shown in Figure 2A. The structural overlay of the models generated in Figure 2A shows that the proposed and conserved residues “SYL” in human STRA6 (red) and zebrafish Rbpr2 (green) are part of an extracellular loop that likely play a critical role in their interaction with circulatory ROL bound to RBP4 (Figure 2A,B and Figure S1). These models were further used in docking studies to analyze the RBP4-STRA6 and RBP4-Rbpr2 interactions by employing the online data-driven docking program HADDOCK2.2 [38,39]. Docking results show that RBP4 interacts with STRA6 and zebrafish Rbpr2 with comparable energy of binding (Table 1; Figure 2B; Figure S1).

### 3.3. Rbpr2 Mutants Encompassing the Proposed RBP4 Binding Domain in Rbpr2 Show Normal Membrane Trafficking

**Figure 3:**
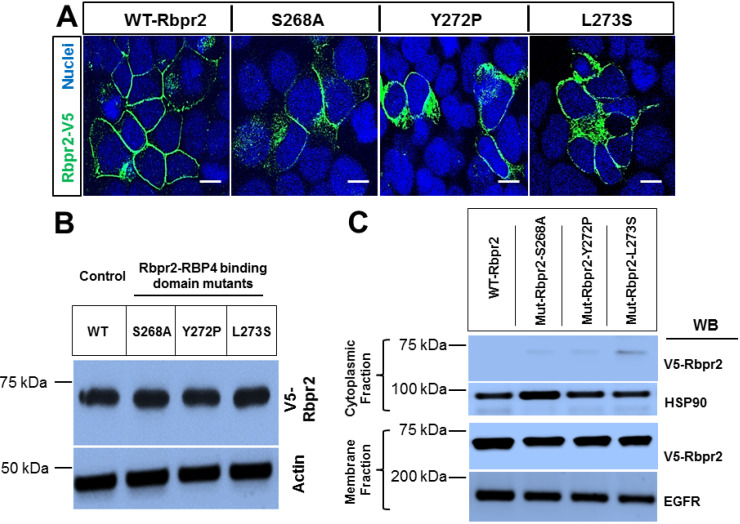
Analysis of expression patterns and membrane trafficking capabilities of zebrafish Rbpr2 mutants affecting the proposed RBP4 binding domain. (**A**) Staining of zebrafish Rbpr2-RBP4 binding domain variants in NIH3T3 cells to examine membrane expression patterns using the V5-antibody. Nucleus, DAPI, blue; Rbpr2-V5, Green. Scale bar = 50 µm. (**B**) Representative Western blot images of WT-Rbpr2 and Rbpr2-RBP4 binding domain mutants. Anti-V5 antibody was used to detect the V5 tagged proteins. Anti-actin was used as the protein loading control. (**C**) NIH3T3 cells expressing either WT-Rbpr2 or individual Rbpr2-RBP4 binding residue mutants were fractionated using the Thermo-Fisher Scientific Subcellular Protein Fractionation Kit. Normalized portions of each extract (~30 µg) were analyzed by Western blotting using specific antibodies against proteins from cytoplasmic (HSP90) and plasma membrane (EGFR). Approximately 50–70 cells from 4–5 fields were imaged and counted per experiment. Each experiment was repeated thrice.

To further study the importance of the RBP4 binding residues on Rbpr2, we used the online line server STRUM (https://zhanglab.ccmb.med.umich.edu/STRUM/), which calculates the change in free energy, ΔΔG, and the fold stability change between the native and mutant protein states. Computational and structural analysis predicted that disruption of any of the individual “SYL” RBP4 binding residues present in the extracellular loop of Rbpr2, would reduce the free energy gap (ΔΔG) between the mutant protein state and its extracellular ligand (Figure 2C,D). Physiologically this is predicted to affect proper cell surface interaction of Rbpr2 with its extracellular ligand RBP4, critical for ROL transport into cells. To test this hypothesis, using site-directed mutagenesis we altered individually the three amino acid residues of the proposed RBP4 ligand-binding sites in zebrafish Rbpr2. The mutagenesis protocol was optimized to generate individual Rbpr2 mutants using the mammalian wild-type (WT) Rbpr2-pCDNA3.1-V5-tagged vector as the template, as previously achieved [32,33,40]. Based on a previous well-established mutagenesis approach for STRA6, the WT polar amino acids (Ser268 and Tyr272) were mutated to hydrophobic amino acids (Ser268Ala and Tyr272Pro), while the hydrophobic amino acid (Leu273) was mutated to a polar amino acid (Leu273Ser) [9,10]. After confirming proper construction of each Rbpr2 mutant plasmid by sequencing, individual Rbpr2 mutants were transfected into NIH3T3 cells, and 72 h post transfection, WT and Rbpr2 mutant expressing cells were subjected to both Western blot analysis and immunostaining using the V5-antibody. Confocal microscopy analyses revealed that like WT-Rbpr2 protein, all three single Rbpr2 binding residue mutants, expressed and trafficked to the plasma membrane in NIH3T3 cells (Figure 3A; green = V5-tagged Rbpr2). Western blot and densitometry analysis further revealed that like WT-Rbpr2, all three single Rbpr2 mutants were equally expressed (Figure 3B; quantified in Figure S2). To confirm the specific subcellular localization of WT and mutant protein, we subjected the WT and the individual Rbpr2 mutant expressing cells to subcellular fractionation, as previously achieved by us [40]. This analysis confirmed that Rbpr2 mutants like the WT-Rbpr2 trafficked to the cell membrane, with the individual Rbpr2 mutants showing minimal cytoplasmic retention (< 5% of total fractionated protein) (Figure 3C; quantified in Figure S2).

### 3.4. Rbpr2 Mutants Encompassing the Proposed RBP4 Binding Domain are Defective in [^3^H]ROL-RBP4 Uptake

**Figure 4:**
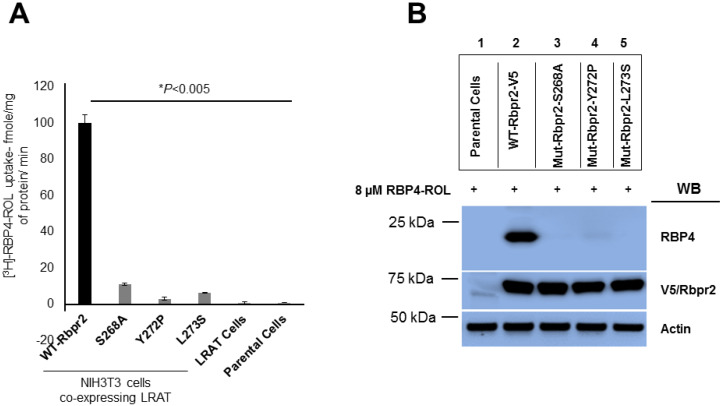
Assessment of RBP4-ROL uptake capabilities of Rbpr2 mutants and Rbpr2–RBP4 protein interaction using co-immunoprecipitation assays. (**A**) Retinol [^3^H]ROL-RBP4 uptake assays in NIH3T3 cells expressing either WT-Rbpr2 or individual Rbpr2 mutants. Although mutants showed normal membrane trafficking capabilities (Figure 3), they showed significant (* *p* < 0.005) decreased ability to take up extracellular RBP4-ROL. (**B**) Stable NIH3T3 cells expressing either the V5-tagged WT-Rbpr2 plasmid (lane 2) or the individual V5-tagged SYL-Rbpr2 mutant plasmid (lanes 3–5) were treated with 8 µM RBP4-ROL for 60 min. Cells were washed thrice to remove any free RBP4-ROL and total protein from cells was isolated. Following anti-V5 immunoprecipitation (IP) using the Pierce Co-IP kit, co-precipitated RBP4 was revealed by anti-RBP4 immunoblotting. Lysate/Input: Portion of total protein lysate post-transfection and prior to IP experiments. Each experiment was repeated thrice. For Figure 4A, statistical analysis of data using the Mann–Whitney *U* test showed a *p* < 0.05 (medians are provided as Box-Whisker plots in Supplementary Figure S13).

To determine whether Rbpr2 mediated RBP4-ROL uptake and subsequent transport into cells is defective in cell lines expressing single Rbpr2 binding residue mutants, we generated WT-Rbpr2 and individual Rbpr2 mutant stable cell lines and then incubated the different stable cell lines with [^3^H]ROL-RBP4. As previously achieved, we analyzed their ability to uptake ROL bound RBP4 at the 60 min time point by scintillation counting [33]. Parental NIH3T3 and NIH3T3/LRAT only expressing cells served as controls. This analysis showed that control cells had no ability to uptake ROL from RBP4 (Figure 4A). However, ROL bound to its plasma protein carrier ([^3^H]ROL-RBP4) was identified in cells expressing WT-Rbpr2, a process that was significantly enhanced in cells expressing both the transporter WT-Rbpr2 and the enzyme LRAT, which catalyzes the transfer of the acyl group from the sn-1 position of phosphatidylcholine to retinol producing retinyl esters, the tissue storage form of dietary vitamin A [3,4,31] (Figure 4A). In contrast, all single Rbpr2 mutant expressing cells showed significantly reduced ability (< 12% activity of WT-Rbpr2) to uptake [^3^H]ROL-RBP4 (Figure 4A).

### 3.5. Co-Immunoprecipitation Assays Show that Rbpr2 mutants Encompassing the Proposed RBP4 Binding Domain have Defective RBP4-ROL Binding Capabilities

Based on structural modeling (Figure 2), with decreased vitamin A uptake capabilities (Figure 4A) but proper membrane trafficking of mutant protein (Figure 3A,C), disruption of the proposed RBP4 binding residues in Rbpr2 likely causes defective extracellular RBP4 binding/interaction for ROL transport. To test this hypothesis, we performed Co-IP experiments with exogenous RBP4 protein in stable NIH3T3/LRAT cells expressing V5-tagged WT-Rbpr2 or individual Rbpr2 mutants. For Co-IP experiments, stable cells were seeded and on reaching 70–80% confluence were washed with PBS to remove any serum and then placed in reduced serum medium (OptiMEM). Then, 8 µM RBP4 was added to the OptiMEM medium of the mutant or control cells for 60 min. Post incubation, cells were collected, washed, protein isolated and subjected to Co-IP (co-immunoprecipitation) analysis using the anti-V5 antibody, followed by Western blotting for RBP4. The Co-IP analysis revealed that while WT-Rbpr2 efficiently bound circulatory RBP4 (Figure 4B, Lane 2; quantified in Figure S2), in contrast each of the three Rbpr2 mutants showed significantly reduced or no ability (> 98% decreased binding capabilities compared to WT-Rbpr2) to bind extracellular RBP4, when correcting for reduced membrane targeting (Figure 4B, Lanes 3, 4 and 5; quantified in Figure S2). Since all three Rbpr2 mutants with similar protein expression and normal cell membrane trafficking capabilities, like WT-Rbpr2, lost extracellular RBP4 binding activity (Figure 3 and Figure 4), demonstrates that their loss of vitamin A uptake activities is due to defective extracellular RBP4 binding. Thus, our in vitro findings imply that the amino acids Ser268, Tyr272 and Leu273 in Rbpr2 likely encompass the RBP4 binding domain in the vitamin A transporter Rbpr2 that would be crucial for ROL transport and therefore this hypothesis warrants in vivo investigation.

### 3.6. Generation of rbpr2 Mutant Zebrafish Lines targeting the RBP4 Binding Domain 

Zebrafish has proven to be a suitable model for studying proteins and mechanisms of ocular retinoid homeostasis in the support of visual function [26,33,35,36,52,53,54,55,56,57,58,59,60,61,62,63,64,65,66,67,68,69,70,71]. Therefore, to investigate in vivo the importance of RBP4 binding domain in Rbpr2 for ROL transport to the developing eye, we used the CRIPSR/Cas9 technology to generate *rbpr2* mutant zebrafish lines targeting the proposed RBP4 binding residues (Figure S3A). Using a previously published protocol that used ssODNs and CRISPR/Cas9, we initially attempted to generate single point mutants, targeting the individual RBP4 binding residues on zebrafish Rbpr2 [66]. However, we were unsuccessful using this approach. Therefore, alternatively we targeted a PAM site upstream, which was in close proximity to the “SYL” residues on Rbpr2, in an effort to disrupt this downstream putative Rbp4-ROL binding domain (Figure 1 and Figure S3A). Injection of CRISPRs along with *cas9* mRNA into developing embryos generated five germline *rbpr2* mutant alleles with cutting close to the PAM site: a 5-bp deletion (*rbpr2^muz98^*), a 1-bp deletion (*rbpr2^muz99^*), a 13-bp deletion (*rbpr2^muz100^*), a 5-bp deletion (*rbpr2^muz101^*) and a 6-bp deletion (*rbpr2^muz102^*) in exon 8 (Figure S3B,C). We chose the *rbpr2^muz99^* mutant zebrafish line for all further analysis. The 1bp deletion in this mutant line resulted in a frameshift, and in the downstream disruption of the RBP4 binding residues, resulting in a pre-mature stop codon after the proposed RBP4 functional domain in *Rbpr2* (Figure S4). To negate any CRISPR off-target effects, the confirmed F1 generation heterozygous *rbpr2^fs-muz99^* mutant carriers were outcrossed for two generations with WT-Tg:TαC-GFP [*Tg(-3.2gnat2:EGFP)^ucd1^/Tg(3.2TalphaCP:EGFP)*], a zebrafish line that constitutively expresses soluble GFP under the control of transducin alpha in cone photoreceptor cells only, and with WT-Tg:XOPS-GFP [*Tg(XlRho:EGFP)^fl1^*], a zebrafish line that expresses soluble GFP under the control of Rhodopsin in rod photoreceptor cells only [50]. The F3 generation heterozygous *rbpr2* mutant carriers (annotated as *rbpr2^fs-^*^muz99^;Tg:TαC-GFP and *rbpr2^fs-^*^muz99^;Tg:XOPS-GFP) were re-confirmed by sequencing genomic DNA from fin-clips. To functionally characterize the *rbpr2^fs-^*^muz99^ mutant, we isolated total RNA from 5.5 dpf mutant and WT larvae, and using gene-specific oligonucleotides obtained Rbpr2-cDNA. The mutant and WT-Rbpr2 cDNA’s were individually cloned into an N’terminus-V5 tagged vector (pSF-CMV-NH2-V5-EKT-NcoI (OG91) N-Terminal V5 Tag Plasmid/Oxford Genetics) and transfected into NIH3T3 cells. Approximately, 72 h post transfection, both WT and Rbpr2^fs-muz99^ mutant expressing cells were subjected to immunostaining using the V5-antibody and DAPI. Confocal microscopy analyses revealed that the Rbpr2^fs-muz99^ mutant protein trafficked to the membrane in NIH3T3 cells, with some cytoplasmic retention (Figure S5D; green = V5-tagged Rbpr2). Stable NIH3T3/LRAT/Rbpr2^muz99^ mutant cell lines were then generated and subjected to uptake assays using [^3^H]ROL-RBP4. This analysis showed that the Rbpr2^muz99^ mutant had significantly reduced ability (< 8% activity of NIH3T3/LRAT/WT-Rbpr2 expressing cells) to uptake [^3^H]ROL-RBP4 (Figure S5E). Whole-mount in situ hybridization (WISH) staining for Rbpr2 mRNA expression, and Rbpr2 mRNA quantitative analysis further revealed that *rbpr2* mutants had similar expression levels, as WT siblings at the 3.5 dpf time point (Figure S5A–C).

### 3.7. rbpr2^fs-muz99^ Mutant Zebrafish Show Eye Phenotypes at Late Larval Stages Typically, Associated with Low Ocular Vitamin A Content and Defective Retinoid Signaling

**Figure 5:**
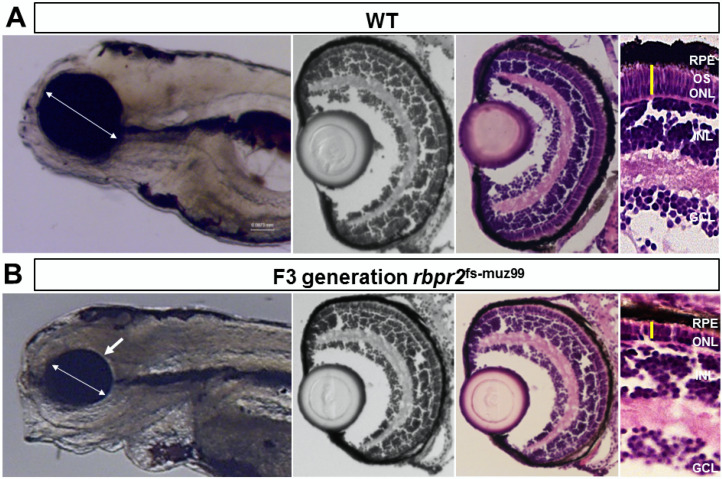
*Rbpr2* mutants affecting the putative RBP4 binding domain show retinal phenotypes. (**A**,**B**) Cross-sections through the eyes of 5.5 dpf WT-Tg:TαC-GFP control (**A**) and F3 generation *rbpr2:*Tg:TαC-GFP homozygous mutant (**B**) larvae. The *rbpr2* mutant eye is smaller (arrow) and shows shorter photoreceptor layer. RPE, retinal pigment epithelium; ONL, outer nuclear layer; INL, inner nuclear layer. At late larval stages mutants showed gross defects, which included hydrocephaly, and pericardial edema. H&E staining of 5.5 dpf WT (**A**) and F3 generation *rbpr2* mutants (*rbpr2*^fs-muz99^; Tg:TαC-GFP) (**B**). For each genotype, approximately 60–80 zebrafish larvae retinas were imaged/analyzed.

The zebrafish yolk contains considerable amounts of maternal vitamin A that must be mobilized to various organs including the eye during development. Herein, a large majority of yolk vitamin A (> 80%) is transported and converted to vitamin A metabolites (retinoids) in the developing eyes, to aid in photoreceptor cell development, survival, and for vision [31,53,57,58]. In support of this hypothesis, it has been shown previously that genetic targeting of vitamin A metabolizing enzymes or transport proteins (Bco2, Bco1, Lrat, Rpe65, Rbp4 or Stra6) in zebrafish provokes a loss of ocular retinoid availability and subsequent signaling during early eye development that results in microphthalmia and retinal phenotypes at larval stages [31,33,57,58,60,61]. Since both Rbp4 and Rbpr2 are expressed during early developmental stages of the zebrafish, disruption of the RBP4 binding domains in Rbpr2 is predicted to significantly affect in vivo Rbpr2 binding to RBP4 and subsequent transport of yolk ROL (in form of RBP4-ROL) to the eye during development [33,59]. Lack of all-*trans* retinol transport to the eye is predicted to result in a decrease of ocular retinoid concentrations/signaling required for proper eye patterning, photoreceptor cell development, and survival [31,32,33,71,72,73,74,75]. To test this hypothesis, we compared eye phenotypes of *rbpr2* mutants to WT siblings at identical ages. Starting at 2 dpf, we observed a smaller eye phenotype in mutant animals (eye size 135 µm ± 5.19 µm) as compared to WT siblings (eye size 165 µm ± 6.32 µm; *p* < 0.05), indicating that loss of Rbpr2 directly affects early eye development (Figure S6). Between the 3 and 4 dpf time-points, the smaller eye phenotype in mutants was still present and more distinguishable, and the animals started to manifest a slight cardiac phenotype (Figure S6). By the 5.5 dpf time-point, eyes were still measurably smaller in homozygous *rbpr2* mutants (eye size 141 µm ± 7.9 µm), compared to WT siblings (eye size 265 µm ± 8.65 µm; *p* < 0.005), and they showed additional systemic phenotypes (hydrocephaly and cardiac edema), indicating that dysfunctional retinoid signaling at late larval stages also affects other organs, as previously reported [14,26,31,57,58] (Figure 5 and Figure S6). Taken together, these results imply that disruption of the RBP4 binding sites in *rbpr2* mutant larvae likely affects in vivo yolk ROL transport to the vertebrate eye during development, resulting in impaired ocular retinoid signaling that manifests in microphthalmia.

### 3.8. rbpr2^fs-muz99^ Mutant Zebrafish Show Retinal Phenotypes at Larval Stages

At the 5.5 dpf time point zebrafish visual system is fully developed and the larva is capable of eliciting a visual response to an external stimulus [52,53,60]. Therefore, using 5.5 dpf as the end-point analysis, retinal histological analysis and immunostaining were performed on WT and *rbpr2* mutant larvae. In transverse sections, all *rbpr2* mutants exhibited smaller eyes (Figure 5 and Figure S6). At 5.5 dpf, although proper retinal lamination was still preserved in mutant eyes, the photoreceptor layers were shorter compared to WT, indicating that proper photoreceptor cell maintenance is dependent on functional Rbpr2 for RBP4-ROL transport to the eye (Figure 5, Figure S6 and S7).

### 3.9. rbpr2^fs-muz99^ Mutant Zebrafish Show Rod and Cone Dystrophy

**Figure 6:**
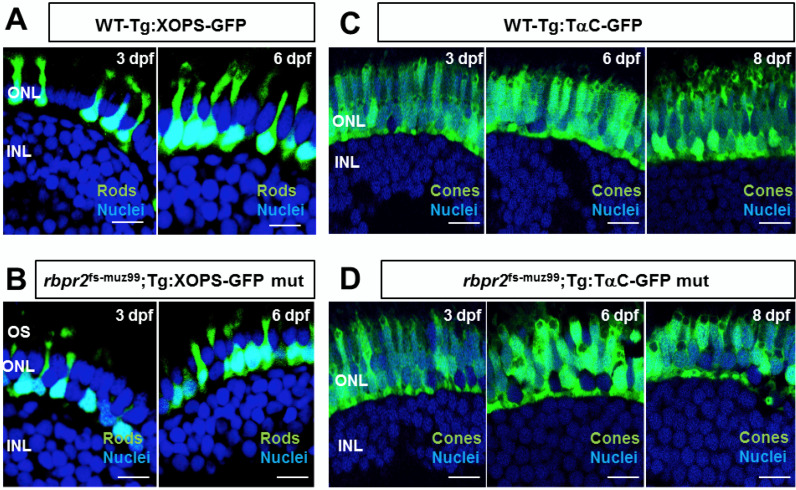
*Rbpr2*^fs-muz99^ mutants affecting the RBP4 binding domain show shorter photoreceptors and progressive degeneration. (**A–D**) Compared to WT-Tg:TαC-GFP zebrafish, *rbpr2* mutants showed shorter rod photoreceptors as early as 3 dpf and loss of cones by 5.5 dpf, evident by decreased soluble GFP expression in both rods and cones of mutants. INL, inner nuclear layer; dpf, days post fertilization. Scale bar = 75 µM. dpf, days post fertilization. For each genotype, approximately 25–30 zebrafish larvae retinas were imaged/analyzed.

Previously, it has been shown that systemic vitamin A deficiency results in sub-optimal ocular retinoid levels and this can deprive the photoreceptor cells of the ocular retinoids, causing disorganization of rod photoreceptor outer segments and progressive degeneration of cone photoreceptor cells [1,26,27,31,32,33,57,64,75,76,77,78]. To test this hypothesis, we used soluble GFP protein expression in transgenic cone and rod lines to determine lengths of photoreceptors (from the photoreceptor synapse to the apical edge of the inner segment) in *rbpr2* mutants, compared to WT-siblings. In addition, we performed HPLC analysis to quantify ocular retinoids in WT and *rbpr2* mutant larvae [50,79]. At the early and late larvae development stages of 3 and 5.5 dpf, respectively, GFP expression was found along the length of rod-photoreceptors in WT zebrafish (Figure 6A). In contrast, based on soluble GFP expression in *rbpr2* mutant’s shorter rod photoreceptors were observed (Figure 6B). In WT siblings, at 3 dpf rod photoreceptor were 9.3 ± 0.22 μm, and at 5.5 dpf, 13.6 ± 0.32 μm in length (*n* = 25 embryos), while *rbpr2* mutant rods were 6.2 ± 0.14 μm, and 5.8 ± 0.29 μm in length (65–75% shorter, *p* < 0.001; *n* = 15 embryos) (quantified in Figure S8). Cone morphology, the predominant photoreceptor cell type in the zebrafish retina was examined next [80,81,82,83,84].

**Figure 7:**
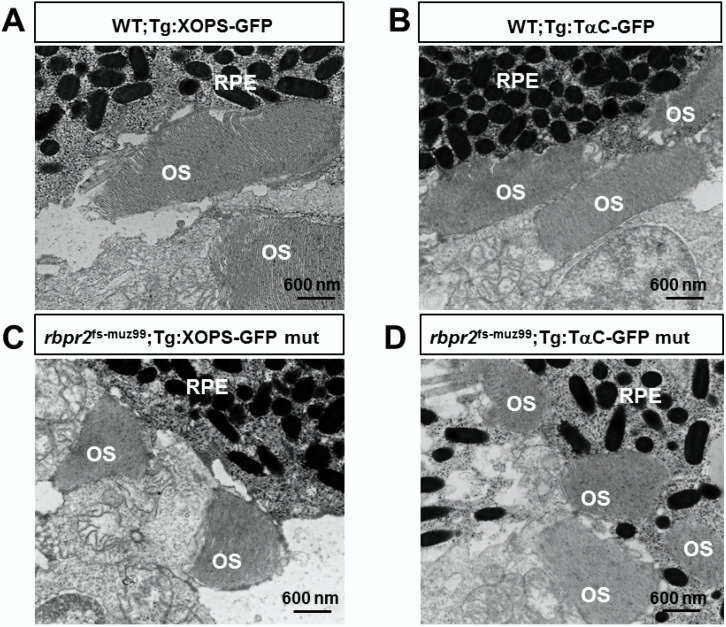
Ultrastructural analysis of *rbpr2*^fs-muz99^ mutant retinas. Transmission electron microscopy provided ultrastructural views of WT-Tg:TαC-GFP and WT-Tg:XOPS-GFP (**A**,**B**) and *rbpr2*^fs-muz99^ mutant (**C**,**D**) photoreceptor cells at 5.5 dpf. Scale bar = 600nm. OS, outer segments; RPE, Retinal Pigmented Epithelium. For each genotype, approximately 25–30 zebrafish larvae retinas were analyzed by EM.

Interestingly, constitutive transducin α-driven soluble GFP expression in cones revealed that at 3 dpf the *rbpr2* mutant cones were similar in size (8.73 ± 0.152 μm) and number compared to their counter parts in WT animals (8.54 ± 0.25 μm; *p* < 0.05). However, in 5.5 dpf *rbpr2* mutants cones were significantly shorter (7.57 ± 0.19 μm), compared to their counterparts in WT animals (14.12 ± 0.19 μm; *p* < 0.001) as shown in Figure 6C vs. Figure 6D (quantified in Figure S8). Shorter photoreceptor outer segments (OS) were observed by transmission electron microscopy (TEM) of *rbpr2* mutant larvae at 5.5 dpf. In both WT and mutant animals, rod and cone photoreceptor OS contained stacks of ordered membranous discs and extended towards the retina pigmented epithelium (RPE) in a parallel and organized manner (Figure 7A,B). However, the photoreceptor OS in *rbpr2^fs-^*^muz99^ mutants were significantly shorter in length (Figure 7C,D).

Counter staining for cones (in *rbpr2^fs-^*^muz99^;Tg:XOPS-GFP mutants) using R/G cone opsin antibody followed by Alexa-594, and for Long-cones (L-cones) in *rbpr2^fs-^*^muz99^;Tg:TαC-GFP mutants using 1D4 antibody followed by Alexa 594, further confirmed shorter cone photoreceptor cells in *rbpr2^fs-^*^muz99^ mutant animals (Figure S9). Such abnormalities were never observed in retinal sections of WT siblings (Figure 6, Figure 7 and Figure S9). Taken together, these results indicate that eye and retinal cell development along with photoreceptor OS survival likely requires a functional RBP4 binding domain in Rbpr2 for ROL transport in the support of proper photoreceptor cell development and maintenance.

### 3.10. rbpr2^fs-muz99^ Mutant Zebrafish show Decreased Ocular Retinoid Content and Signaling 

**Figure 8:**
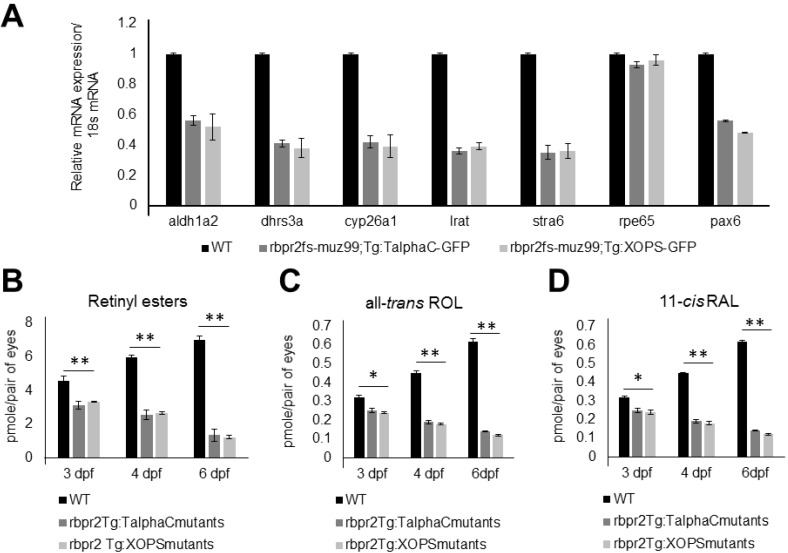
Downregulation of retinoid signaling regulated genes and reduced retinoid content in *rbpr2*^fs-muz99^ mutant zebrafish eyes. (**A**) Retina-specific gene expression dependent on all-*trans* retinoic acid (*at*RA) signaling were compared by qPCR using equal amounts of total RNA from heads of wild-type/control (black bars) and *rbpr2* mutants (grey bars) at 3.5 dpf. *aldh1a2*, *dhrs3a*, *cyp26a1*, *lrat, stra6, pax6* and *rpe65* mRNA expression were normalized to 18S ribosomal RNA. mRNA expression values of genes in controls (WT animals) were set to 1, and difference in gene expression between the two genotypes are shown as relative fold change normalized to endogenous 18S RNA. * *p* < 0.001. (**B**,**C**,**D**) Retinoid content of zebrafish heads representing retinoid composition of eyes of control WT (*n* = 25; black bars) and pooled *rbpr2*^fs-muz99^; Tg:TαC-GFP or *rbpr2*^fs-muz99^;Tg:XOPS-GFP mutant (*n* = 15 each genotype; grey bars) animals at 3, 4 and 6 dpf. *, *p* < 0.05., **, *p* < 0.005. Each q-RTPCR experiment was repeated twice using freshly prepared cDNA. For Figure 8B–D statistical analysis of data using the Mann–Whitney *U* test showed a *p* < 0.05 (medians are provided as Box-Whisker plots in Supplementary Figure S14).

Gross eye pathology, retinal histology, and rod-cone photoreceptor dystrophy observed in *rbpr2*^fs-muz99^ mutants suggested that loss of Rbpr2 likely affects yolk vitamin A transport to the eye resulting in decreased ocular retinoid production [26,31,57,58,79]. To test this hypothesis, we analyzed ocular retinoid content of *rbpr2*^fs-muz99^ mutants (*n* = 25) and WT/controls (*n* = 25) by HPLC and used Q-PCR to evaluate mRNA expression of genes that are known to be induced by retinoid (all-*trans* retinoic acid) signaling. This analysis revealed significantly decreased expression levels of *aldh1a2* (3.6-fold decrease; *p* < 0.005), encoding a retinaldehyde dehydrogenase that converts retinaldehyde into all-*trans* RA; *dhrs3a* (5.8-fold decrease; *p* < 0.005), which encodes a dehydrogenase that reduces the amount of retinaldehyde available for conversion to all-*trans* RA, *cyp26a1* (3.4-fold decrease; *p* < 0.005), which encodes an enzyme that catabolizes all-*trans* RA to non-biological metabolites, *lrat* (3.9-fold decrease; *p* < 0.005), the enzyme which produces *all-trans* retinyl esters, and *stra6* (3.8-fold decrease; *p* < 0.005) a retinoic acid inducible gene, in mutants as compared to WT siblings, indicating sub-optimal levels of ocular retinoic acid in mutants (Figure 8A and Figure S10; WT, black bars; *rbpr2* mutants, dark and light grey bars).

Since retinoid production is in part controlled by a*t*ROL availability [17,18,27,32,33,53,55,61,64,68,69,77,80,81,82,83,84,85,86,87,88,89], this quantitative gene expression analysis data strongly suggests that *rbpr2^fs-^*^muz99^ mutants have decreased levels of all-*trans* retinol and subsequent metabolites (including a*t*RA) in the eyes, as compared to WT siblings. Finally, to confirm this hypothesis, and that loss of ocular retinoid signaling was the cause of photoreceptor degeneration in *rbpr2^fs-^*^muz99^ mutant animals, we determined the retinoid contents in heads of 3, 4 and 6 dpf larvae by quantitative HPLC analyses. This analysis showed a 9–14-fold reduction of 11-*cis* retinaldehyde (11-*cis* RAL), all-*trans* retinol (all-*trans* ROL), and retinyl esters (RE) in *rbpr2^fs-^*^muz99^ mutants, compared to WT siblings at similar age (*p* < 0.005; Figure 8B–D).

### 3.11. Apoptosis Pattern of Retinas in rbpr2^fs-muz99^ Mutant Zebrafish

**Figure 9:**
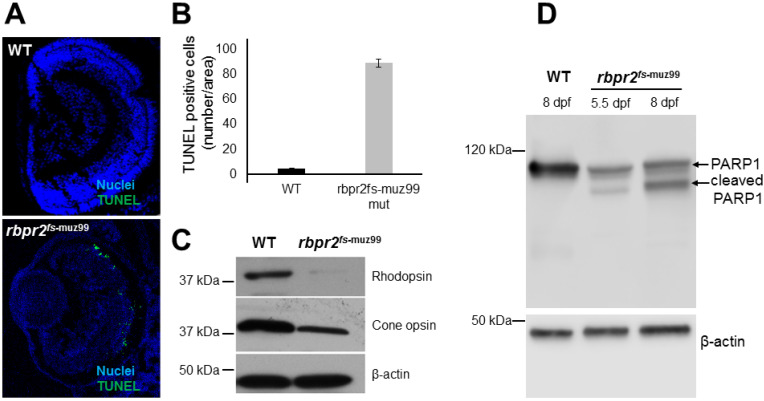
Detection of apoptosis in retinas of *rbpr2*^fs-muz99^ mutants. (**A**) Wild-type (WT) and *rbpr2* mutant (*rbpr2*^fs-muz99^) zebrafish retinas at 5.5 dpf were stained with TUNEL reagent. The TUNEL positive cells/apoptotic nuclei stain green. (**B**) Quantification of TUNEL positive cells. (**C**) Determination of rod and cone opsin levels in WT and mutant eyes (dissected heads; *n* = 12 each genotype) by Western blot analysis at 8 dpf. (**D**) Total protein from WT and mutant eyes (dissected heads; *n* = 12 each genotype), at the 5.5 and 8 dpf time-points, were pooled and subjected to Western blot analysis using PARP1 antibody. Cleaved PARP1 products in *rbpr2*^fs-muz99^ mutant eyes indicate apoptosis. TUNEL assays and Western blot experiments were repeated thrice. For Figure 9B, statistical analysis of data using the Mann–Whitney *U* test showed a *p* < 0.05 (medians are provided as Box-Whisker plots in Supplementary Figure S15).

Apoptosis is a common feature of photoreceptor cell death in conditions of impaired retinoid signaling [33,55]. We therefore tested for apoptosis in the retinas of *rbpr2^fs-^*^muz99^ mutants (*n* = 25) and WT/controls (*n* = 25), by Terminal deoxynucleotidyl transferase (TdT) dUTP Nick-End Labeling (TUNEL) assay, designed to detect apoptotic cells that undergo extensive DNA degradation during the late stages of apoptosis. In 5.5 dpf *rbpr2^fs-^*^muz99^ mutant zebrafish, apoptotic-positive signals were detected in particular in the outer nuclear layer (ONL) of the central and peripheral retina (Figure 9A; quantified in Figure 9B).

To further investigate this mechanism, we isolated protein from the heads of 5.5 and 8 dpf old *rbpr2^fs-muz99^* mutant and WT zebrafish (*n* = 12 each genotype and time point) and performed Western blot analysis for opsins and for cleaved PARP1. This analysis showed a significant decrease of both rod and cone opsins in *rbpr2^fs-muz99^* mutant zebrafish, compared to WT animals, likely reflecting the shorter photoreceptors observed in immunofluorescence and TEM images (Figure 6, Figure 7 and Figure 9C). This analysis further showed presence of cleaved PARP1 in *rbpr2*^muz99^ mutants (Figure 9D). Since, PARP1 helps cells to maintain their viability, detection of PARP1 cleavage indicated that retinal cells in *rbpr2^fs-muz99^* mutants were undergoing apoptosis at late larval stages (Figure 9D). Taken together, these data in zebrafish demonstrate that transport of yolk-derived ROL to the eye for proper ocular retinoid production/signaling is dependent on Rbpr2 function. 

### 3.12. WT-Rbpr2 mRNA and All-trans Retinoic Acid (atRA) rescue the rbpr2^fs-muz99^ Mutant Phenotype

**Figure 10:**
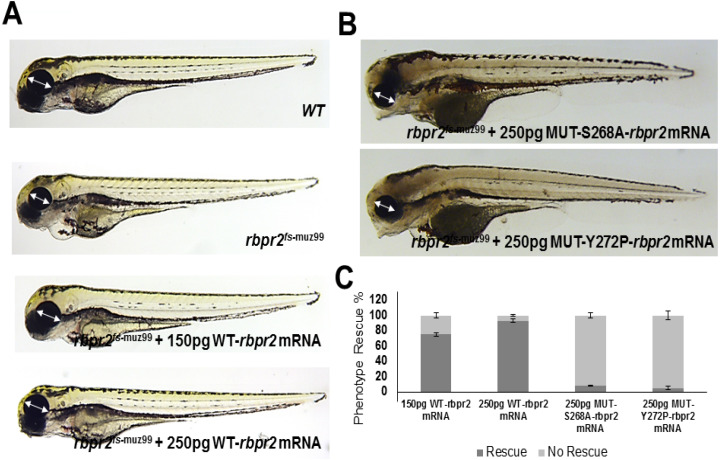
WT-*rbpr2* mRNA rescues the *rbpr2*^fs-muz99^ mutant phenotype. (**A**) Rescue experiments were performed by injecting WT zebrafish *rbpr2* mRNA into control and *rbpr2*^fs-^*^muz^*^99^ *mutant* embryos at the 1–2 cell stage. Using light microscopy, at 5.5 dpf, low dose (150 pg) reconstitution of *rbpr2* mRNA in *rbpr2^muz^*^99^ mutant embryos partially rescues the eye phenotype while high dose (250 pg) *rbpr2* mRNA fully rescues the *rbpr2*^fs-^*^muz^*^99^ mutant phenotype. (**B**) Rescue experiments were performed by injecting 250pg of mutant zebrafish *rbpr2* mRNA (S268A or Y272P) into control and *rbpr2*^fs-^*^muz^*^99^ *mutant* embryos at the 1–2 cell stage. Using light microscopy at 5.5 dpf, reconstitution of mutant *rbpr2* mRNA in *rbpr2*^fs-muz99^ embryos failed to rescue the eye phenotype. (**C**) Quantification of *rbpr2*^fs-muz99^ phenotype rescue from Figure 10A,B. For each genotype, approximately 45–50 zebrafish larvae were imaged/analyzed. Rescue experiments were repeated twice using freshly prepared mRNA. For Figure 10C, statistical analysis of data using the Mann–Whitney *U* test showed a *p* < 0.05 (medians are provided as Box-Whisker plots in Supplementary Figure S16).

**Figure 11:**
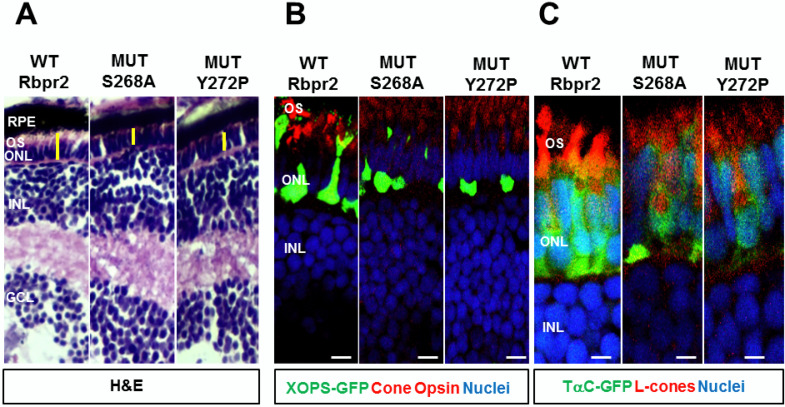
Histological analysis and immunostaining of *rbpr2*^fs-muz99^ mutant larvae eyes after WT or mutant Rbpr2 mRNA injection. (**A**) Histological analysis and H&E staining, (**B**) immunostaining for R/G cone opsins, and (**C**) immunostaining for L-cones (1D4 antibody) of Tg:*rbpr2*^fs-muz99^ mutant larvae eyes at 5.5 dpf, after WT-*rbpr2* mRNA or mutant-*rbpr2* mRNA injection. Scale bar = 50 µM (**B**); Scale bar = 25 µM (**C**). For each genotype, approximately 25–30 zebrafish larvae retinas were images/analyzed.

To confirm that the defects observed in *rbpr2^fs-muz^*^99^ mutants were caused specifically by loss of RBP4 binding residues in Rbpr2, we performed rescue experiments by injecting wild-type (WT) zebrafish *rbpr2* mRNA into control and *rbpr2* mutant embryos at the 1–2 cell stage. At 5.5 dpf, we found that low dose (150 pg) reconstitution of WT-*rbpr2* mRNA, in *rbpr2* mutant embryos, partially rescues the heart and eye phenotype while high dose (250 pg) WT-*rbpr2* *mRNA* fully rescues the *rbpr2* mutant phenotype (Figure 10A and Figure 11; quantified in Figure 10C). In contrast, Rbpr2 ligand-binding mutants *(S268* and *Y272P) mRNA* were unable to efficiently rescue the eye phenotypes in *rbpr2^fs-muz99^* mutants. (Figure 10B and Figure 11; quantified in Figure 10C). Rescue experiments were also performed with the all-*trans* ROL metabolite, all-*trans* retinoic acid (a*t*RA). Exogenously applied a*t*RA was dissolved in DMSO and applied to the fish water containing embryos at the 40% epiboly stage, just before gastrulation begins.

At 5 dpf, low dose a*t*RA (0.2 uM) treatment resulted in a partial rescue of the mutant phenotype but a higher dose a*t*RA (0.5 uM) treatment resulted in a complete rescue of the mutant phenotype (Figure S11A–C). To further test the importance of the RBP4 binding domains in Rbpr2 for ROL transport during eye development, we obtained another mutant line from the Zebrafish International Resource Center (ZIRC: *rbpr2*^sa32616^). The mutation in this line occurs in exon 13 of the zebrafish *rbpr2* gene but “after” the proposed RBP4 binding domain and is predicted to result in a pre-mature stop codon (Figure 1 and Figure S12). Analysis of the *rbpr2*^sa32616^ mutant larvae at 5.5 dpf by light microscopy and retinal histology showed no eye phenotypes, with the exception of a slight bent/curved tail (Figure S12). Taken together, these results indicate that the “SYL” residues on Rbpr2 likely encompasses the functional RBP4-ROL binding domain that is necessary for ROL transport to the vertebrate eye for proper photoreceptor cell development and survival.

## 4. Discussion

In this study, we generated a global KO zebrafish model of Rbpr2, a vitamin A/all-*trans* retinol/ROL transporter, by specifically targeting (upstream) a proposed RBP4-retinol (RBP4-ROL) binding domain that is partially conserved among species. This animal model, with a targeted disruption of the RBP4-ROL binding sites, phenocopied retinal cell phenotypes in humans seen with vitamin A deficiency, including presentation of microphthalmia, shorter rod and cone photoreceptor OS, decreased ocular retinoid content, with late systemic phenotypes of cardiac abnormalities and hydrocephaly. The current study focused on the role of Rbpr2 in RBP4 binding and all-*trans* retinol transport from the zebrafish yolk to the eye for ocular retinoid production in the support of photoreceptor cell maintenance and survival. The loss of the presumptive RBP4-ROL binding domain in *rbpr2* mutant zebrafish lines first manifested in a smaller eye phenotype at early developmental stages accompanied with degeneration of rod and cone photoreceptor cells at late larval stages. These phenotypes were linked to the lack of ocular retinoid content likely caused by defective RBP4 binding and ROL transport to the eye in *rbpr2* mutant animals during development. We hypothesize that the vitamin A transporter, Rbpr2, contains a functional RBP4-ROL binding domain in this region, necessary for yolk vitamin A transport to the eye during development, to aid in proper eye patterning, photoreceptor cell maintenance and function in the support of vision. Limitations to this approach in identifying the putative Rbp4 binding sites in zebrafish Rbpr2 are discussed below (Section 4.6).

### 4.1. Importance of Dietary Vitamin A Transporters for Vision

Evolution’s choice of dietary vitamin A as a precursor for the vital signaling molecule (all-*trans* retinoic acid/RA) for retinal cell development and maintenance, and the essential visual chromophore (11-*cis* retinaldehyde/RAL) in photoreceptors, triggered selective pressure to advance an efficient system of transporters for dietary vitamin A/all-*trans* retinol/ROL uptake and storage in the continuous support of vision [5,6,7,28,29,30,31,32,33,59,86,90]. All-*trans* retinol (ROL) is the main transport form of dietary vitamin A in the blood [5,11,12]. During transport virtually all ROL is bound to plasma retinol-binding protein (RBP4). RBP4 delivers ROL from the liver, the main organ of storage, to distant organs that need vitamin A, such as the eyes, brain, lungs, kidneys, placenta and other peripheral organs [5,6,16]. Therefore, the RBP4-ROL delivery, together with RBP4 membrane receptor system, by distributing stored vitamin A to the eye, helps vertebrates maintain photoreceptor cell structure and function for vision [5,16,29]. Whether this system also plays a functional role during vertebrate retinal cell development had yet to be investigated.

### 4.2. Importance of RBP4-ROL Binding Residues in Vitamin A Receptors 

Previously, biochemical and genetic studies from the Sun and von Lintig laboratories have implicated the STRA6 gene product in the cellular uptake of dietary vitamin A bound to RBP4 in the eye [28,30]. Complementary to this mechanism, we recently showed in zebrafish that the second retinol binding protein receptor 2 (Rbpr2), which shares structural homology with STRA6, is expressed in peripheral tissues, can bind to RBP4, and can thereby facilitate systemic ROL transport to the eye and other organs. Loss of Rbpr2 in mutant zebrafish lines resulted in a loss of retinoid signaling during embryogenesis manifesting in retinal phenotypes at larval stages [32,33]. Since both vitamin A receptors bind RBP4-ROL, this suggests that they must both contain a functional RBP4 binding domain critical for ROL transport. Previously, the Sun laboratory, using site-directed mutagenesis, biochemical studies and in vitro vitamin A uptake assays identified putative functional/ligand-binding residues in the extracellular loop of STRA6 for RBP4 binding and vitamin A uptake [9,10]. Complementary to this, genetic studies from the von Lintig group using *Stra6* KO animals, showed that loss of STRA6 leads to vitamin A deficiency in the eyes, manifesting in severe retinal phenotypes [29,30]. It is also interesting to note that the RBP4 binding domain residues in zebrafish Rbpr2 lie within a previously proposed RBP4-ROL binding domain in human STRA6, identified using in vitro site mutagenesis and vitamin A uptake assays in HEK293 cells [9,10,28,43] (Figure 1A). However, the existence of a potential RBP4 binding domain in the systemic vitamin A transporter Rbpr2 has yet to be characterized in vivo. 

### 4.3. Rbpr2 Contains a Putative RBP4-ROL Binding Domain Important for Yolk Vitamin A Transport to the Eye

In this study, we used the zebrafish model and eye phenotypes as functional read outs for proper ROL transport during development, to further study the structure and function of the vitamin A transporter Rbpr2 for systemic retinol uptake from RBP4. In our first approach, we used homology modeling, molecular docking analysis, site-directed mutagenesis and [^3^H]ROL-RBP4 uptake assays to first confirm in vitro the importance and functionality of the proposed RBP4-ROL binding domains in Rbpr2 [2,9,10]. Alignment and comparison of proposed RBP4-ROL binding domains among different species showed conserved amino acid domains within human STRA6 and Rbpr2. The proposed S268, Y272 and L273 amino acid residues in Rbpr2 are also partially conserved in STRA6 and are part of a previously proposed binding domain/loop [9,10]. Mutagenesis of any of these three amino acid residues in Rbpr2 was predicted to cause changes in the free energy gap leading to a potential for loss of extracellular RBP4-ROL binding and transport. Indeed, even while individual SYL mutants in cultured cells showed predominant cell membrane trafficking capabilities, all three mutants (individually) showed almost no ability to bind and transport circulatory RBP4-ROL. Thus, as all three mutants with normal cell membrane trafficking lost extracellular RBP4 binding capability demonstrates that their loss of vitamin A uptake activities is due to defective RBP4 binding. These in vitro data therefore demonstrate the specific interaction between RBP4 and Rbpr2 and confirmed the importance of the S268, Y272 and L273 “SYL” amino acid residues in the vitamin A receptor Rbpr2 for RBP4-ROL binding and transport.

### 4.4. Stra6 and Rbpr2

Rbpr2 shares structural homology with STRA6 and exhibits comparable RBP4-binding and retinol uptake kinetics [2,9,10,30,33,43]. Therefore, Rbpr2 appears to be a previously unrecognized member of the STRA6 family. Computer simulations predict that RBPR2 has a structure related to that of STRA6, with 9 to 11 predicted transmembrane segments. It also shares conserved amino acids with STRA6 which are essential for either the structure or function of these proteins. However, Rbpr2 and Stra6 differ in several ways. Rbpr2 and Stra6 exhibit fundamentally different patterns of tissue expression [2,30,31,32,33]. Stra6 is not expressed in liver or intestine, tissues where RBPR2 is most highly expressed. RBPR2 lacks the STRA6 C’ terminus Src homology 2 domain recently found to mediate RBP4-dependent Jak-STAT signaling in certain cell types [5]. All-*trans* Retinoic acid (a*t*RA) suppresses RBPR2 expression, but has been known to up-regulate STRA6 [2,30]. Because a*t*RA production is in part controlled by all-*trans* retinol availability, opposite regulation of the two proteins by a*t*RA could be a mechanism for directing RBP4 or retinol flux as needed either to liver (expressing Rbpr2 when circulating RBP4, retinol, and/or intracellular a*t*RA levels are low) or to extrahepatic tissues, like the eye (expressing STRA6 when RBP4, all-*trans* retinol, and/or a*t*RA are high). Future studies in mouse models are needed to determine whether inverse regulation of RBPR2 and STRA6 by a*t*RA plays a role in coordinating in vivo retinol homeostasis.

### 4.5. RBP4 Binding Residues on Rbpr2 are Necessary for Systemic Vitamin A Transport

Previously, it has been suggested that >80% maternal retinoids present in the yolk of the zebrafish embryo is metabolized and transported specifically to the vertebrate eye to aid in eye and retinal cell development during larval stages. [31,49,57,58,61]. Herein, the ROL transport system, via retinol binding protein 4 (as circulatory RBP4-ROL), is proposed to specifically function in the ocular vitamin A-dependent processes of vision in zebrafish [29,30,58,61], mice [11,51,64], and humans [5,77,90]. In the mammalian embryo, ROL is provided from the maternal circulation via the placenta during organ development, whereas avian, reptilian, amphibian and fish embryos use retinoid stores from the egg yolk [31,52,57,58]. In the zebrafish, these retinoids including ROL are presumed to be transported from the yolk stores to aid in embryonic eye formation, patterning, and establishment of the visual system. Therefore, membrane receptors for ROL transport must already be present and functional to support the uptake and transport of retinoids to the developing embryonic eye [51]. Supporting this hypothesis, we and others have shown that both Rbp4, Stra6, and Rbpr2 are expressed at early developmental stages in zebrafish likely to support maternal yolk ROL transport to the developing vertebrate eye [31,32,33,59]. Therefore, to test in vivo the functionality of the “SYL” RBP4 binding residues on Rbpr2 for ROL transport we established a mutant zebrafish line targeting specifically the proposed three amino acid RBP4-ROL binding residues. In fact, analysis of phenotypes in *rbpr2* mutant zebrafish showed early manifestations of smaller eyes starting at the 2 dpf time point and this phenotype remained consistent till 5.5 dpf (latest time point of analysis). Subsequently to the 3 dpf time point, cardiac and brain phenotypes were visible which have been previously related to defects in general retinoid metabolism. HPLC analysis for ocular retinoids and quantitative qPCR analysis for retinoic acid responsive genes in the retinas of *rbpr2^fs-muz99^* mutants at these time points showed a progressive decrease in levels of ocular retinoid content and loss of retinoid/a*t*RA signaling. Interestingly, these observations and results are consistent with those seen in RP and LCA patients whom harbor genetic alterations in retinoid cycle genes and show progressive cone photoreceptor cell degeneration due to depleted ocular retinoids levels [16,19,73].

Among organs, the eye is most frequently affected in animal models of vitamin A deficiency [14,18,20,21,26,27,30,31,32,33,54,56,68,69]. Our findings here are also consistent with this pattern. Likewise, in humans, total loss of *RBP4* is only associated with night blindness, retinal dystrophy and chorioretinal coloboma [64]. Given the central role of retinoids in light perception, this unique sensitivity is striking and may reflect an evolutionary origin of RA signaling in the visual system [12,21]. The hypothesis that disruption of RBP4 binding residues on *Rbpr2* affects RBP4-ROL binding and uptake invariably diminishing ocular retinoid content/signaling was tested by performing rescue experiments with all-*trans* RA (a*t*RA) and by measuring expression of ocular genes associated with vitamin A metabolism (retinoid metabolism) and a*t*RA signaling. a*t*RA treatment of *rbpr2*^fs-muz99^ mutant zebrafish rescued the mutant phenotype in a dose-dependent manner, indicating that decreased levels of the all-*trans* ROL metabolite a*t*RA affects cellular signaling in the mutants (Figure S12).

### 4.6. Limitations of the rbpr2 mutant in Identifying “SYL” Residues as the Putative Rbp4 Binding Domain

The main zebrafish mutant (*rbpr2^fs-^*^muz99^) used as an argument to show SYL function (Rbp4 binding domain) in vivo has a frameshift upstream of the domain of interest on Rbpr2, which leads to the generation of a premature stop codon and a protein-containing ~280 aa. The observed retinal phenotype in vivo could therefore be caused by knockout of the Rbpr2 protein and not specifically by a loss of the SYL binding domain. Thus, it is possible that the “SYL” binding domain is not the only Rbp4 binding sequence that could exist on Rbpr2. Therefore, to test this possibility, we are currently generating point mutant *Rbpr2* knock-in mutant mice, targeting the individual “SYL” RBP4 binding residues.

### 4.7. Mechanism(s) of Photoreceptor Cell Death in rbpr2 ^fs-muz99^ Mutants

Optimal levels of the ligand (11-*cis* retinal) are required during rhodopsin synthesis for successful signaling and that without 11-*cis* retinal, photoreceptors may degenerate [15,23]. Additionally, Rhodopsin is crucial for photoreceptor OS development and survival since mice lacking rhodopsin do not develop OS and photoreceptors degenerate [71,80]. Subsequently, decreased rhodopsin, cone opsins, detection of TUNEL positive nuclei in retinas of *rbpr2^fs-muz99^* mutants and cleavage of PARP1 products, further suggested that cell death occurred likely due to impaired ocular retinoid levels, establishing the importance of Rbpr2 in yolk ROL transport to the eye for retinoid production in proper eye development, photoreceptor OS maintenance, and survival.

## 5. Conclusions

For dietary vitamin A to reach the eye it must first be systemically absorbed. Distribution of maternal or dietary derived vitamin A throughout the body is important to maintain retinoid function in peripheral tissues and to ensure optimal vision. A critical step of this process is the transport of vitamin A across cell membranes. This study first identified in vitro a proposed critical RBP4 binding domain in the second RBP4-ROL transporter, Rbpr2, which is expressed in systemic organs. Because mutations in all three residues of the proposed RBP4 domain decimated the RBP4 binding and vitamin A transport activities of Rbpr2, while showing proper cell membrane trafficking, suggests that these residues are likely located in the contact site for the Rbpr2-RBP4 interaction. Our in vivo study using mutant zebrafish further confirmed the importance of the RBP4 binding domain in Rbpr2 for ROL transport to developing organs. Zebrafish mutants targeting the RBP4 binding domain showed microphthalmia and retinal cell development phenotypes at early larval stages, followed by systemic phenotypes at larval stages. The observations that WT-Rbpr2, and not mutant Rbpr2 was capable of rescuing the mutant eye phenotype, suggests the importance of these RBP4 binding residues on Rbpr2 for yolk ROL transport. These results show the need for the vitamin A receptor Rbpr2 in RBP4 binding and transport of maternal yolk retinol during vertebrate eye development and for photoreceptor cell survival. The study of Rbpr2 in rod and cone photoreceptor cell maintenance also highlights the likely importance of this vitamin A transporter in late stage larvae for sustaining ROL transport to the retina for photoreceptor health, function and survival. Thus, modulation of such vitamin A transporters for improving vision could be tested in the future using such animal models. The generality of our findings in cell culture and zebrafish models as presented here therefore requires further testing in mammalian models such as *Rbpr2* knockout mutants and/or CRISPR-Cas9 generated point mutation *Rbpr2* knock-in mice specifically targeting the individual RBP4-ROL binding residues.

## Figures and Tables

**Table 1:** Parameters obtained using the online docking server HADDOCK for RBP4-Rbpr2 and RBP4-STRA6 interactions.

	RBP4-Rbpr2 Docking	RBP4-STRA6 Docking
HADDOCK score	−85.6 +/− 2.0	−81.4 +/− 3.1
RMSD from the overall lowest-energy structure	0.6 +/− 0.3	16.0 +/− 0.3
Van der Waals energy	−33.3 +/− 1.5	−39.8 +/− 1.8
Electrostatic energy	−67.2 +/− 14.1	−16.6 +/− 2.0
Desolvation energy	−41.6 +/− 1.9	−38.7 +/− 3.5
Restraints violation energy	27.3 +/− 13.23	3.8 +/− 2.54
Buried Surface Area	1127.8 +/− 74.6	902.8 +/− 35.0
Z-Score	–1.6	–1.4

## Supplementary Materials

The following are available online at https://www.mdpi.com/2073-4409/9/5/1099/s1, Figure S1: Surface filled view of Rbpr2 and RBP4 protein-protein interactions based on HADDOCK docking analysis, Figure S2: Densitometry analysis of western blots, Figure S3: Generation of *rbpr2-*RBP4 binding domain zebrafish mutants, Figure S4: Functional consequences of the CRISPR/Cas9 generated *rbpr2^fs-muz99^* mutant zebrafish line, Figure S5: Functional characterization of the *rbpr2*^fs-muz99^ mutant, Figure S6: Manifestation of early eye phenotypes in *rbpr2*-RBP4^fs-muz99^ mutants, Figure S7: Retinal phenotypes of *rbpr2*^fs-muz99^;Tg:XOPS-GFP mutant animals at 5.5 dpf, Figure S8: Quantification of photoreceptor length in *rbpr2*^fs-muz99^ mutant animals, Figure S9: Counterstaining in transgenic *rbpr2*^fs-muz99^ mutants, Figure S10: Downregulation of retinoid signaling regulated genes in *rbpr2*^fs-muz99^ mutant zebrafish eyes, Figure S11: All-*trans* retinoic acid rescues the *rbpr2*^fs-muz99^ mutant phenotype, Figure S12: ZIRC *rbpr2* mutants (*rbpr2*^sa32616^) encompassing a mutation after the proposed RBP4 binding sites in Rbpr2 do not show eye phenotypes, Figure S13: Quantification and Statistical analysis of Retinol uptake in cells of data in Figure 4A, Figure S14: Retinoid content of zebrafish heads representing retinoid composition of eyes of control WT and pooled *rbpr2*^fs-muz99^ mutants of data in Figure 8B–D, Figure S15: Quantification and Statistical analysis of TUNEL positive cells of data in Figure 9B, Figure S16: Quantification and statistical analysis of *rbpr2*^fs-muz99^ phenotype rescue of data in Figure 10A,B.
