# Supplementary material for: A Functional Binding Domain in the Rbpr2 Receptor Is Required for Vitamin A Transport, Ocular Retinoid Homeostasis, and Photoreceptor Cell Survival in Zebrafish
Source: Cells. 2020 Apr 29;9(5):1099. doi: 10.3390/cells9051099 (PMC7290320; doi:10.3390/cells9051099)
Supplement: Supplementary file 1 [file cells-09-01099-s001.pdf]

SUPPLEMENTAL INFORMATION

A functional binding domain in the Rbpr2 receptor is required for vitamin A transport, ocular retinoid homeostasis, and photoreceptor cell survival in zebrafish

Ashish K. Solanki[1], Altaf A. Kondkar[2], Joseph Fogerty[3], Yanhui Su[1], Seok-hyung Kim[1], Joshua H. Lipschutz[1,4], Deepak Nihalani[1], Brian D. Perkins[3] and Glenn P. Lobo[1,5*].

[1] Department of Medicine, Medical University of South Carolina, Charleston, SC, 29425, USA. lobo@musc.edu (G.P.L.); solankia@musc.edu (A.K.S.); su@musc.edu (Y.S.); nihalani@musc.edu (D.N.); lipschut@musc.edu (J.H.L.); kims@musc.edu (S.H.K.).
[2] Glaucoma Research Chair, Department of Ophthalmology, College of Medicine, King Saud University, Riyadh, Saudi Arabia. akondkar@ksu.edu.sa (A.A.K.)
[3] Department of Ophthalmic Research, Cole Eye Institute, Cleveland Clinic, Cleveland, OH 44195, USA. fogertj@ccf.org (J.F.); perkinB2@ccf.org (B.D.P.).
[4] Ralph H. Johnson VA Medical Center, Division of Research, Charleston, SC 29420, USA. lipschut@musc.edu (J.H.L.).
[5] Department of Ophthalmology, Medical University of South Carolina, Charleston, SC, 29425, USA. lobo@musc.edu (G.P.L.)
* Correspondence: lobo@musc.edu; Tel.: 843-876-2371

***Corresponding Author**

Glenn P. Lobo, Ph.D.
Assistant Professor of Medicine
Department of Medicine
Drug Discovery Building DDB513
70 President Road
Charleston, SC 29425
Office: 843-876-2371
E-mail: lobo@musc.edu

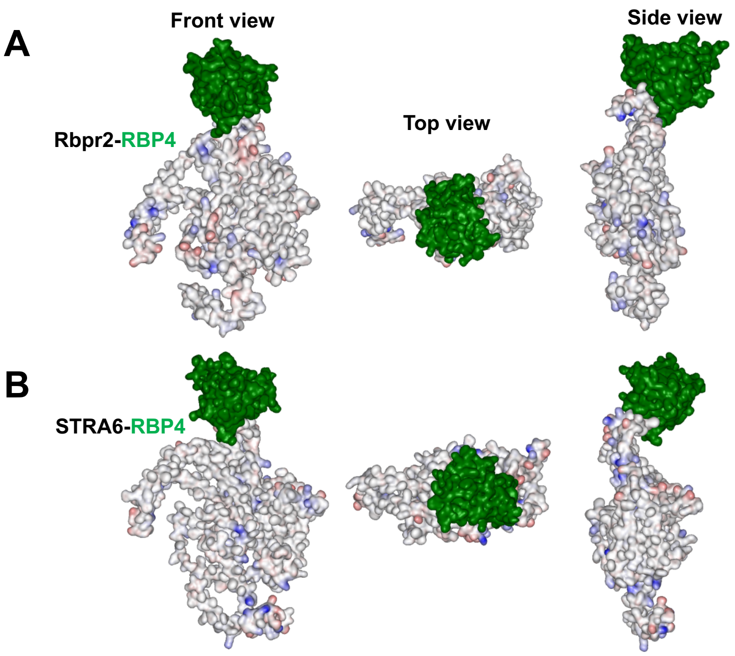

Figure S1. Surface filled view of Rbpr2 and RBP4 protein-protein interactions based on HADDOCK docking analysis.

Multiple surface filled views of (**A**) Zebrafish Rbpr2 with human RBP4, and (**B**) Zebrafish Stra6 with Human RBP4, protein-protein interactions are shown.

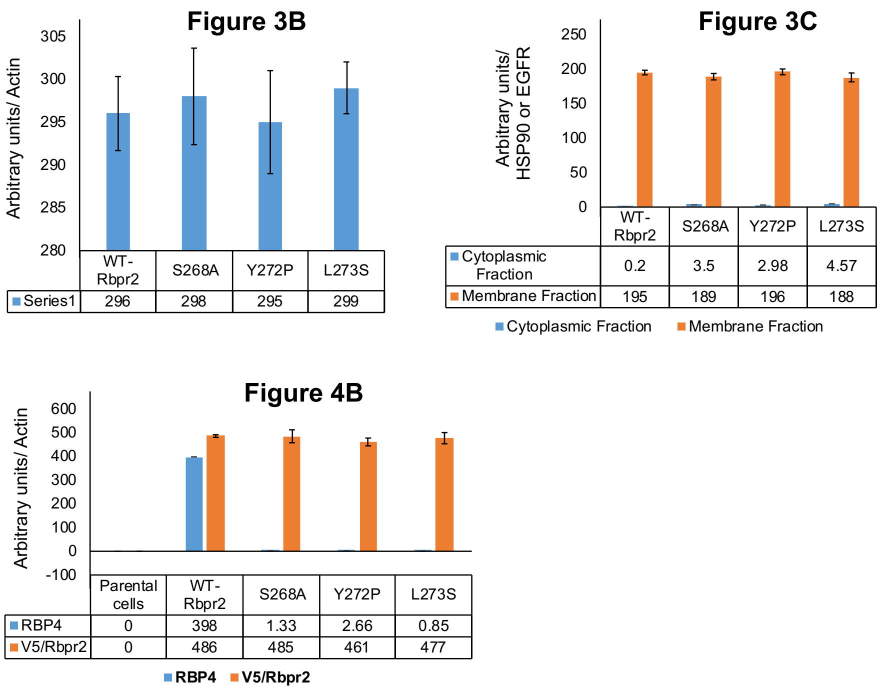

Figure S2. Densitometry analysis of western blots.

Image J software was used to quantify (Arbitrary Units) protein bands from Figure 3B, 3C, and 4B. Analysis is representative from three western blot experiments.

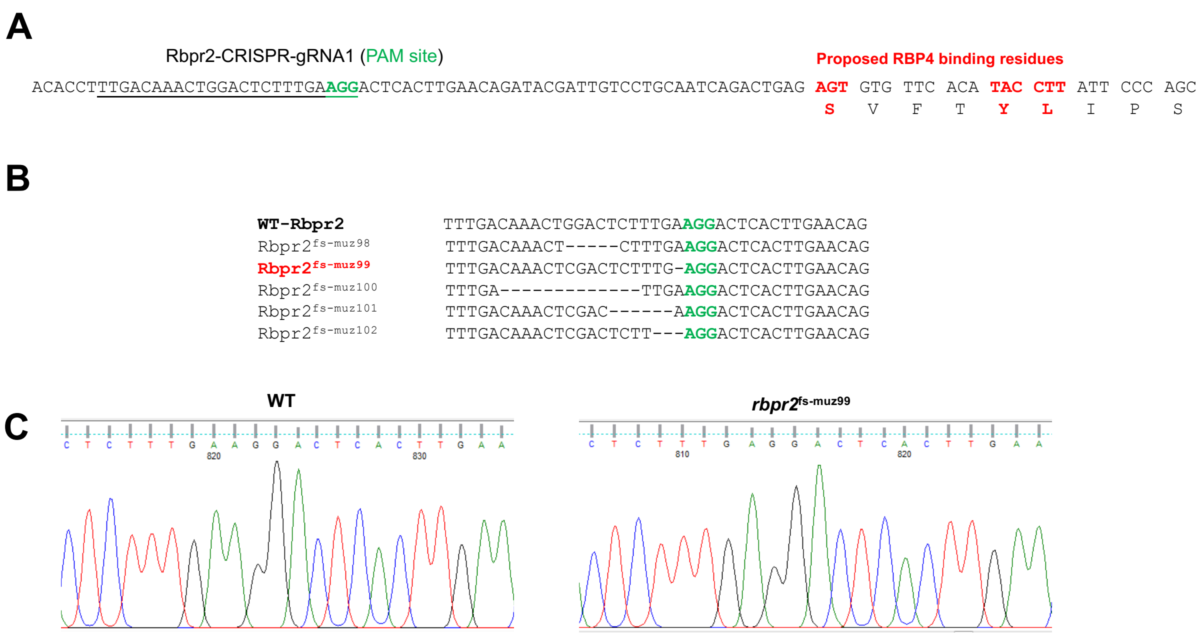

Figure S3. Generation of *rbpr2*-RBP4 binding domain zebrafish mutants.

(**A**) The proposed RBP4 binding domain in zebrafish Rbpr2 was targeted using CRISPR gRNAs (underlined), PAM site shown in green. (**B**) The CRISPR/Cas9 cutting generated multiple mutant alleles. A 1-bp deletion in the zebrafish Rbpr2 (*rbpr2*$^{\text{fs-muz99}}$) coding sequence that results in a frameshift that affects the downstream SYL-RBP4 binding domain was chosen for all further analysis. (**C**) Sequencing chromatograms from wild-type (WT) and *rbpr2*$^{\text{fs-muz99}}$ mutant.

```
                              CRISPR/Cas9 "PAM" site
WT-Rbpr2  ACC  TTT  GAC  AAA  CTG  GAC  TCT  TTG  AAG  GAC  TCA  CTT  GAA  CAG  ATT  GCA  TTG  TCC  TGC
           T    F    D    K    L    D    S    L    K    D    S    L    E    Q    I    A    L    S    C
          245  246  247  248  249  250  251  252  253  254  255  256  257  258  259  260  261  262  263

                                                *delA
Rbpr2muz99 ACC  TTT  GAC  AAA  CTG  GAC  TCT  TTG*AGG  ACT  CAC  TTG  AAC  AGA  TTG  CAT  TGT  CCT  GCA
           T    F    D    K    L    D    S    L    R    T    H    L    N    R    L    H    C    P    A
          245  246  247  248  249  250  251  252  253  254  255  256  257  258  259  260  261  262  263


                    "SYL" proposed RBP4 binding residues

WT-Rbpr2  AAT  CAG  ACT  GAG  AGT  GTG  TTC  ACA  TAC  CTT  ATT  CCC  AGC  ATC  AAT  ATG  AGT  TCA  GCA
           N    Q    T    E    S    V    F    T    Y    L    I    P    S    I    N    M    S    S    A
          264  265  266  267  268  269  270  271  272  273  274  275  276  277  278  279  280  281  282

Rbpr2muz99 ATC  AGA  CTG  AGA  GTG  TGT  TCA  CAT  ACC  TTA  TTC  CCA  GCA  TCA  ATA  TGA  GTT  CAG  CAT
           I    R    L    R    V    C    S    H    T    L    F    P    A    S    I   Stop  V    Q    H
          264  265  266  267  268  269  270  271  272  273  274  275  276  277  278  279  280  281  282
```

Figure S4. Functional consequences of the CRISPR/Cas9 generated *rbpr2*$^{fs\text{-}muz99}$ mutant zebrafish line.

The 1bp deletion in this *rbpr2* mutant line resulted in a frameshift, and in the downstream disruption of the RBP4 binding residues, resulting in a pre-mature stop codon after the proposed RBP4 functional domain in Rbpr2.

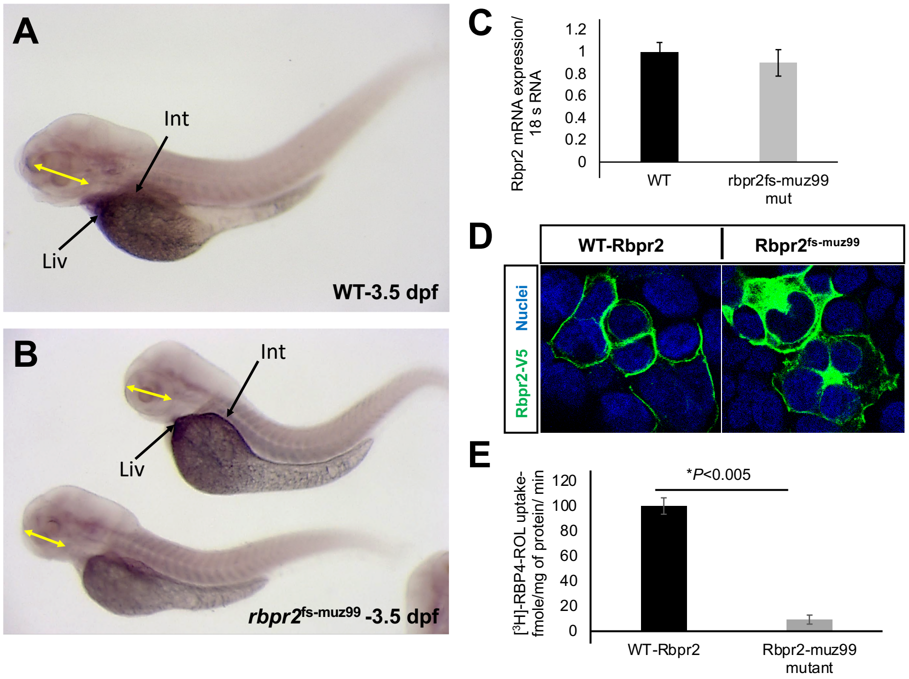

Figure S5. Functional characterization of the *rbpr2*$^{\text{fs-muz99}}$ mutant.

(**A, B**) Rbpr2 mRNA expression patterns in WT-larvae (**A**), and *rbpr2*$^{\text{fs-muz99}}$ mutants (**B**) at 3.5-days post fertilization (dpf) analyzed by whole mount *In-situ* Hybridization (WISH). (**C**) Zebrafish Rbpr2 mRNA expression quantification by Q-RTPCR. (**D**) Functional characterization of the *rbpr2*$^{\text{fs-muz99}}$ mutant by protein localization analysis in NIH3T3 cells. Scale bar=50µm. (**E**) [^{3}H]ROL-RBP4 uptake assays in NIH3T3 cells expressing either WT-Rbpr2 or *rbpr2*$^{\text{fs-muz99}}$ mutant.

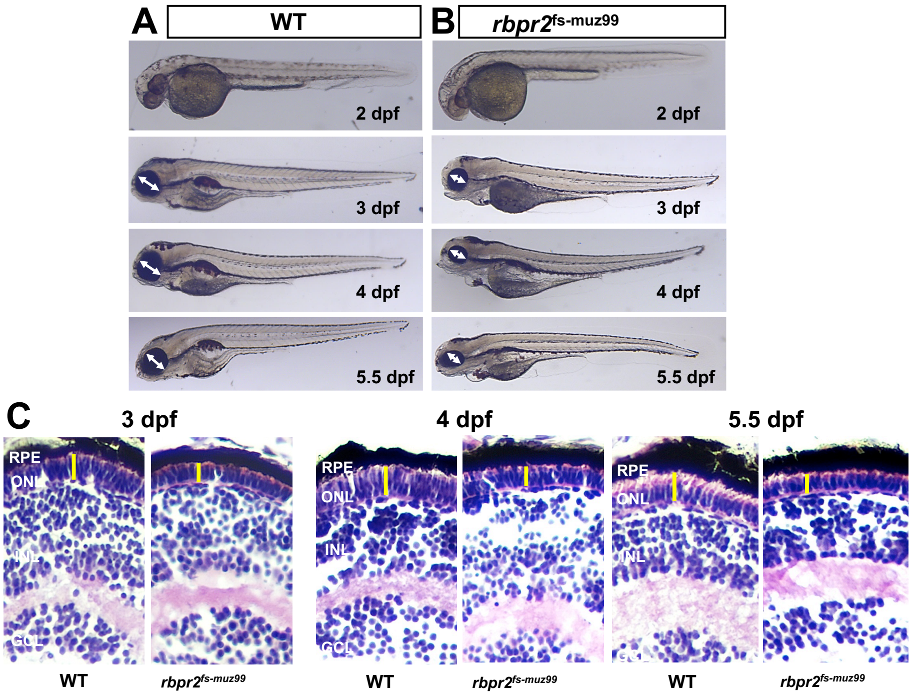

Figure S6.

Manifestation of early eye phenotypes in *rbpr2*-RBP4$^{fs\text{-}muz99}$ mutants.

Compared to WT animals (**A**), loss of Rbpr2 in *rbpr2*-RBP4 mutant animals (**B**) manifests in early eye phenotypes during developmental stages. Systemic phenotypes in *rbpr2*-RBP4 mutant animals is attributable to general defects in retinoid metabolism during late larval stages. (**C**) Retinal histology and H&E staining of retinas from 3, 4 and 5.5 dpf WT and *rbpr2*-mutants. Photoreceptor outer segments (OS) appear shorter as compared to WT retinas at similar development time-points. Retinal lamination layers in mutants, like in WT larvae, were well preserved at the indicated time points of analysis. OS, outer segments; IS, inner segments; ONL, outer nuclear layer; INL, inner nuclear layer; WT, wild-type; dpf, days post fertilization.

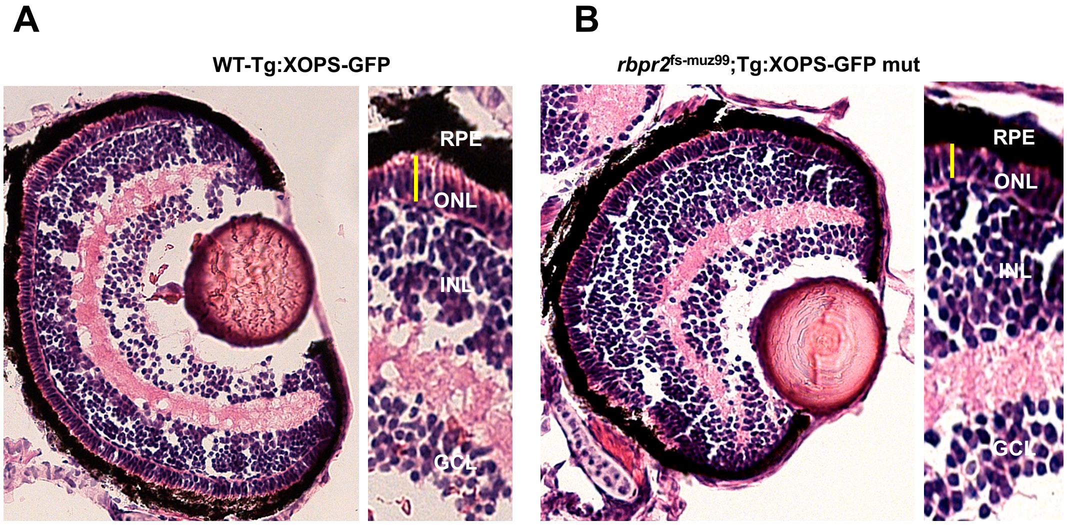

Figure S7.

Retinal phenotypes of *rbpr2*$^{fs\text{-}muz99}$;Tg:XOPS-GFP mutant animals at 5.5 dpf.

Compared to WT-Tg:XOPS-GFP zebrafish (**A**), eyes of F3 generation *rbpr2*$^{fs\text{-}muz99}$;Tg:XOPS-GFP mutant animals (**B**) were smaller, and showed shorter photoreceptor layer outer segments by H&E analysis. ONL, outer nuclear layer; INL, inner nuclear layer; GCL, ganglion cell layer; dpf, days post fertilization; RPE, retina pigmented epithelium.

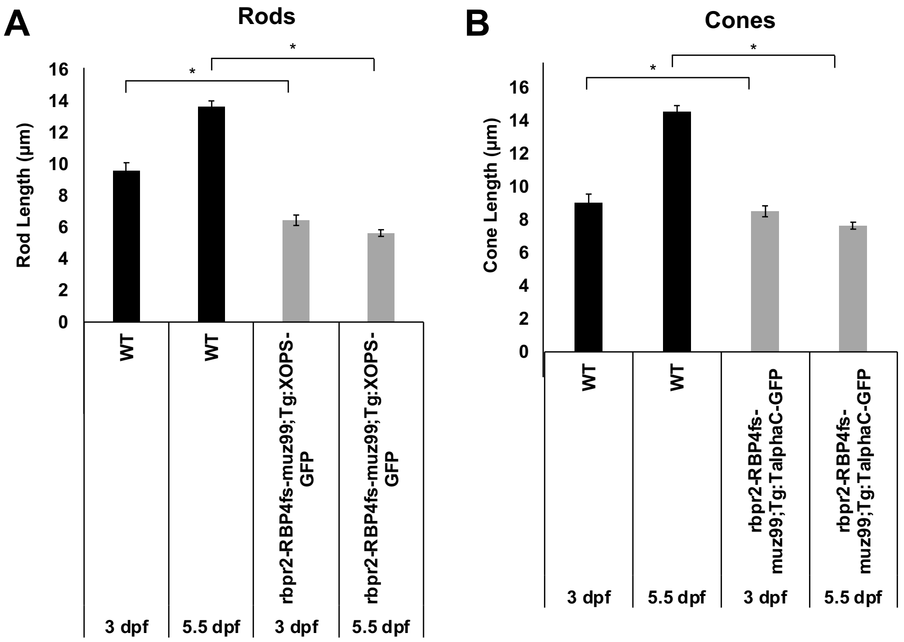

Figure S8.

Quantification of photoreceptor length in *rbpr2*$^{\text{fs-muz99}}$ mutant animals.

Image J was used to quantify and measure GFP staining along the length of the photoreceptors (from the photoreceptor synapse to the apical edge of the inner segment) in rods (**A**) and cones (**B**) in both WT and *rbrp2*-mutant animals at 3 dpf and 5.5 dpf time points. Approximately 150 cones and rods in WT and *rbpr2* mutant retinas were counted and sized.

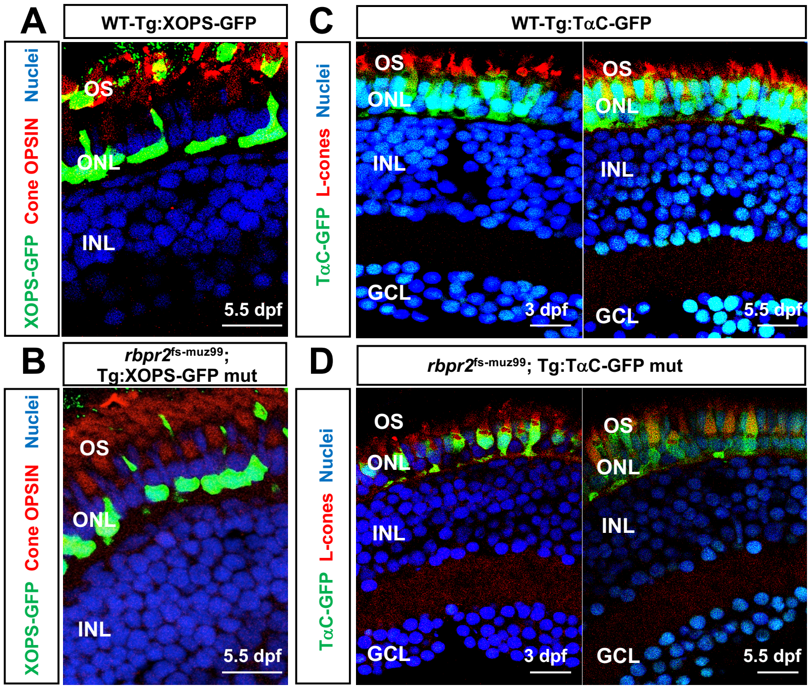

Figure S9.

Counterstaining in transgenic ***rbpr2***$^{fs\text{-}muz99}$ **mutants.** (**A, B**) WT-Tg:XOPS-GFP and *rbpr2*$^{fs\text{-}muz99}$;Tg:XOPS-GFP mutant retinas that express GFP in rods only were counter stained with R/G cone opsin antibody followed by Alexa 594. (**C, D**) WT Tg:TαC-GFP and *rbpr2*$^{fs\text{-}muz99}$;Tg:TαC-GFP mutant retinas that express GFP in cones only were counter stained with 1D4 (L-cones) antibody followed by Alexa 594. Nuclei were stained with DAPI. (**A, B**) scale bar=50μm; (**C, D**) scale bar=75μm. OS, outer segments; INL, Inner nuclear layer; ONL, Outer nuclear layer; dpf, days post fertilization; IS, Inner segments.

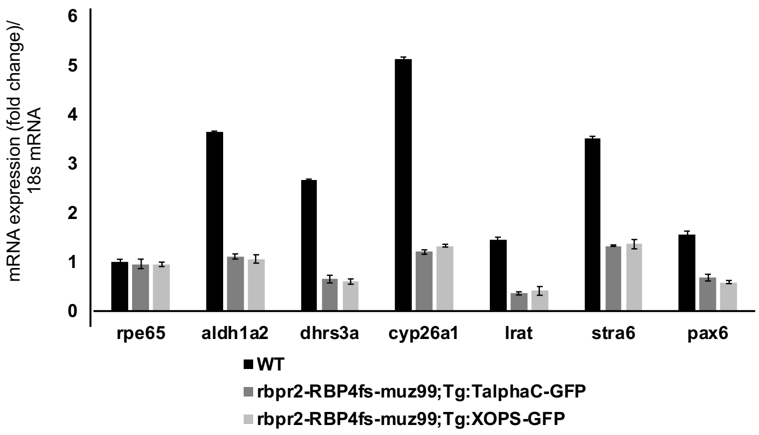

Figure S10.

Downregulation of retinoid signaling regulated genes in *rbpr2*$^{fs\text{-}muz99}$ mutant zebrafish eyes.

Retina-specific gene expression dependent on RA signaling were compared by qPCR using equal amounts of total RNA from heads of wild-type/ control (black bars) and *rbpr2* $^{fs\text{-}muz99}$ mutants (grey bars) at 3.5 dpf. Rpe65 mRNA expression values were set to 1 and difference in gene expression between the two genotypes are shown as relative fold change normalized to endogenous 18S RNA. *p<0.005.

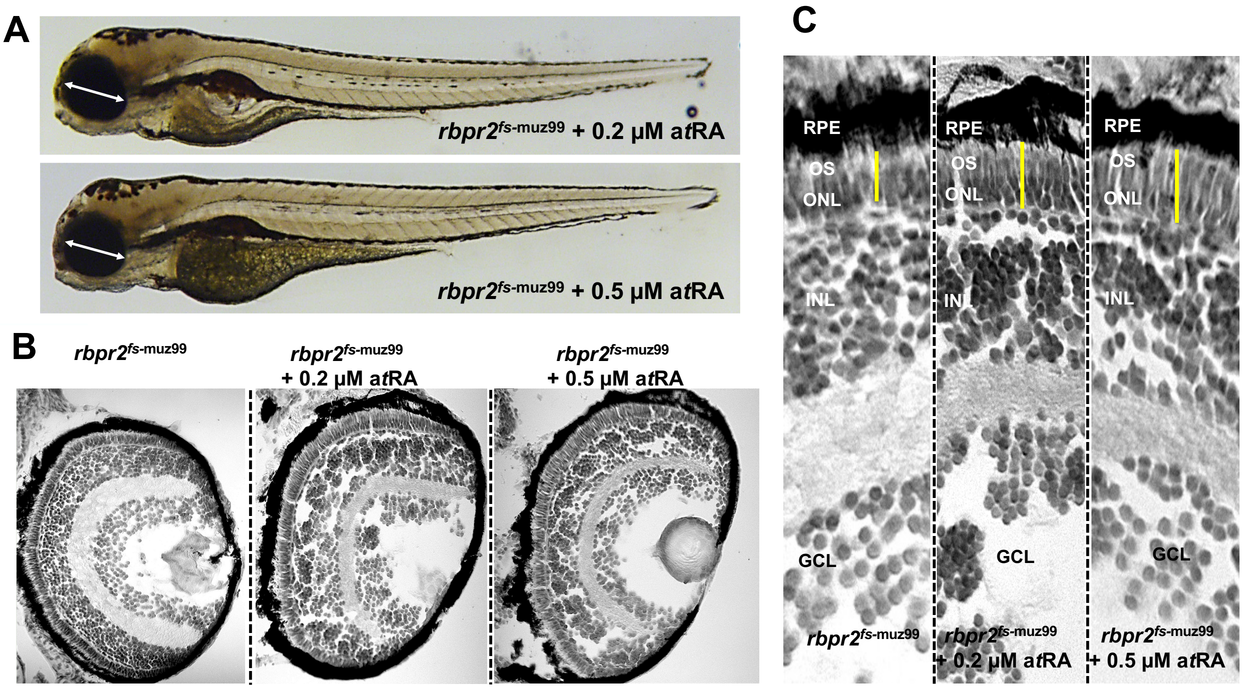

Figure S11.

All-*trans* retinoic acid rescues the *rbpr2*$^{fs\text{-}muz99}$ mutant phenotype.

(**A**) Dose specific treatment with all-*trans* retinoic acid (a*t*RA) rescues the *rbpr2*$^{fs\text{-}muz99}$ mutant phenotype. Images obtained at 5-5.5 dpf. Rescue experiments of *rbpr2* mutants with either mRNA or a*t*RA were repeated twice as outlined in methods. (**B, C**) Representative images of eye sections from *at*RA treated *rbpr2*$^{fs\text{-}muz99}$ mutants at 5.5 dpf. RPE, retinal pigmented epithelium; OS, outer segments; INL, inner nuclear layer; ONL, outer nuclear layer; dpf, days post fertilization.

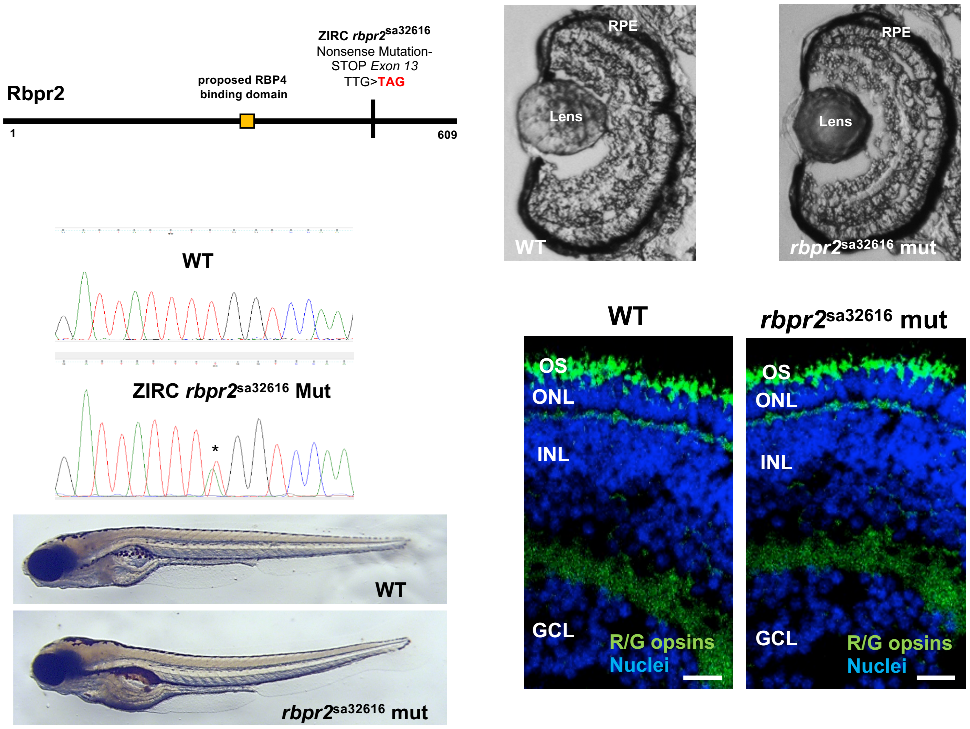

Figure S12.

ZIRC *rbpr2* mutants (*rbpr2*^{sa32616}) encompassing a mutation after the proposed RBP4 binding sites in Rbpr2 do not show eye phenotypes.

A *rbpr2* mutant zebrafish line (G>A mutation; *rbpr2*^{sa32616}) from the Zebrafish International Resource Center (ZIRC) which results in a premature stop codon in exon 13, was obtained and analyzed by light microscopy, histology and immunohistochemistry at 5.5 dpf. With the exception of a curved/ bent tail, no other significant phenotype was observed in this zebrafish *rbpr2*-mutant. Note: The TTG>TAG mutation in exon 13 of the Rbpr2 coding sequence occurs “after” the proposed RBP4 binding sites. Immunostaining for cone photoreceptors (R/G opsins antibody) revealed that cones in both WT and mutants at 5.5 dpf were similar in number and showed normal morphology. OS, outer segments; IS, inner segments; PRL, photoreceptor cell layer; ONL, outer nuclear layer; INL, inner nuclear layer; GCL, ganglion cell layer; WT, wild-type; dpf, days post fertilization.

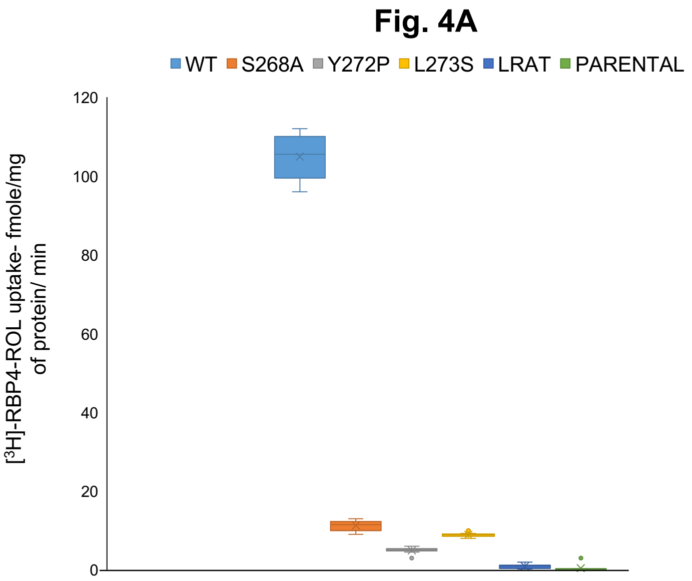

Figure S13. Quantification and Statistical analysis of Retinol uptake in cells of data in Figure 4A.

Retinol [^{3}H]ROL-RBP4 uptake assays in NIH3T3 cells expressing either WT-Rbpr2 or individual Rbpr2-mutants were statistically analyzed using the Mann–Whitney *U* test. The data between groups were significant (P<0.05).

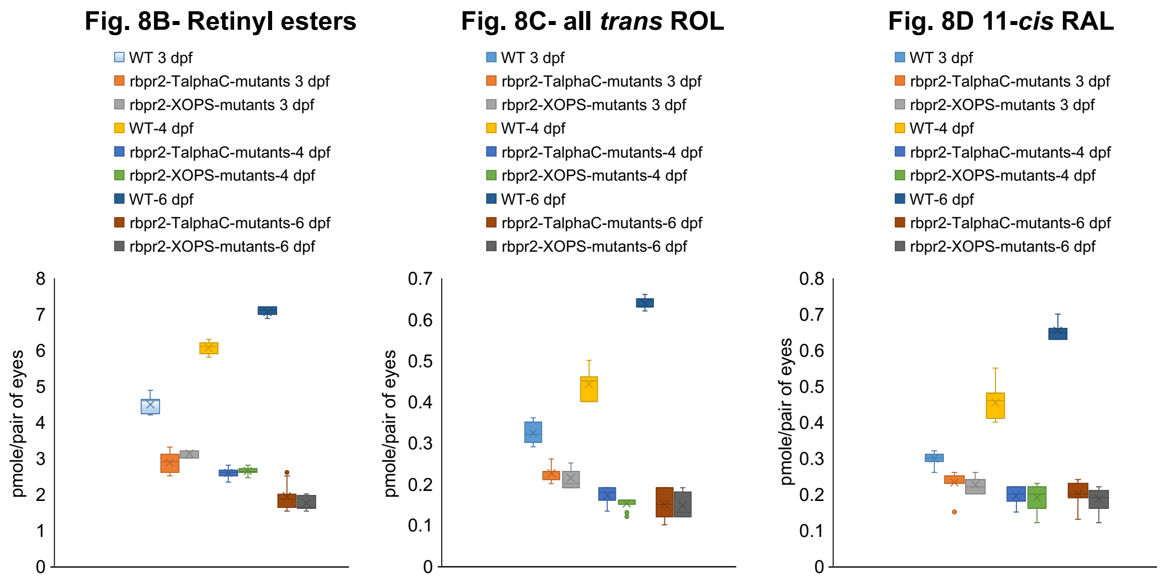

Figure S14. Retinoid content of zebrafish heads representing retinoid composition of eyes of control WT and pooled *rbpr2*$^{fs\text{-}muz99}$ mutants of data in figures 8B-8D. Statistical analysis of differences in retinoid content in animals were analyzed using the Mann–Whitney *U* test. The data between groups were significant ($P<0.05$).

Fig. 9B

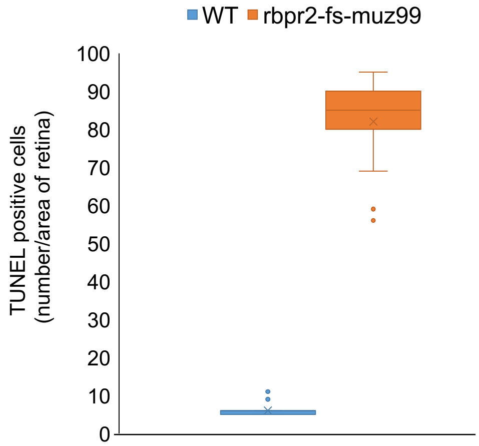

Figure S15. Quantification and Statistical analysis of TUNEL positive cells of data in Figure 9B. Statistical analysis of differences in apoptotic cells in retinas of WT and *rbpr2* mutant animals were analyzed using the Mann–Whitney *U* test. The data between groups were significant (P<0.05).

Fig. 10C

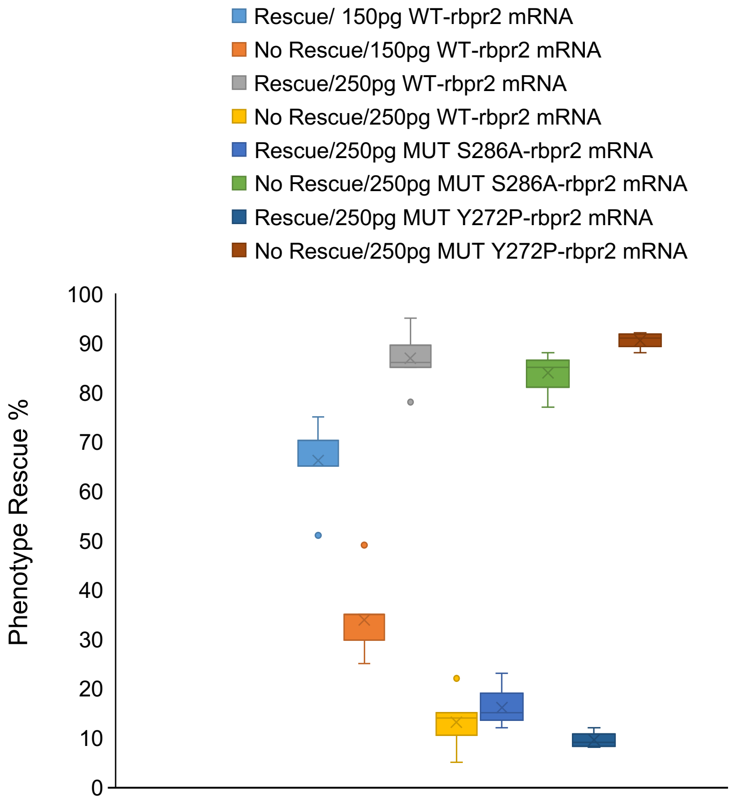

Figure S16. Quantification and statistical analysis of *rbpr2*$^{fs\text{-}muz99}$ phenotype rescue of data in Figures 10A and 10B.

Statistical analysis of differences in rescue of *rbpr2* mutant phenotypes were analyzed using the Mann–Whitney *U* test. The data between groups were significant ($P<0.05$).
